# Microbial adhesion and biofilm formation by *Candida albicans* on 3D-printed denture base resins

**DOI:** 10.1371/journal.pone.0292430

**Published:** 2023-10-04

**Authors:** Marcela Dantas Dias da Silva, Thais Soares Bezerra Santos Nunes, Hamile Emanuella do Carmo Viotto, Sabrina Romão Gonçalves Coelho, Raphael Freitas de Souza, Ana Carolina Pero

**Affiliations:** 1 Department of Dental Materials and Prosthodontics, Araraquara School of Dentistry, São Paulo State University (UNESP), Araraquara, SP, Brazil; 2 Faculty of Dental Medicine and Oral Health Sciences, McGill University, Montreal, QC, Canada; Yerevan State Medical University Named after Mkhitar Heratsi, ARMENIA

## Abstract

This study evaluated surface properties and adhesion/biofilm formation by *Candida albicans* on 3D printed denture base resins used in 3D printing. Disc-shaped specimens (15 mm x 3 mm) of two 3D-printed resins (NextDent Denture 3D+, NE, n = 64; and Cosmos Denture, CO, n = 64) and a heat-polymerized resin (Lucitone 550, LU, control, n = 64) were analyzed for surface roughness (Ra μm) and surface free energy (erg cm^-2^). Microbiologic assays (90-min adhesion and 48-h biofilm formation by *C*. *albicans*) were performed five times in triplicate, with the evaluation of the specimens’ surface for: (i) colony forming units count (CFU/mL), (ii) cellular metabolism (XTT assay), and (iii) fluorescence and thickness of biofilm layers (confocal laser scanning microscopy). Data were analyzed using parametric and nonparametric tests (α = 0.05). LU presented higher surface roughness Ra (0.329±0.076 μm) than NE (0.295±0.056 μm) (p = 0.024), but both were similar to CO (0.315±0.058 μm) (p = 1.000 and p = 0.129, respectively). LU showed lower surface free energy (47.47±2.01 erg cm^-2^) than CO (49.61±1.88 erg cm^-2^) and NE (49.23±2.16 erg cm^-2^) (p<0.001 for both). The CO and NE resins showed greater cellular metabolism (p<0.001) and CO only, showed greater colonization (p = 0.015) by *C*. *albicans* than LU in the 90-min and 48-hour periods. It can be concluded that both 3D-printed denture base resins are more prone to colonization by *C*. *albicans*, and that their surface free energy may be more likely associated with that colonization than their surface roughness.

## Introduction

*Candida albicans* is a yeast-like fungus present in the human body that is frequently related to oral infectious diseases, dubbed oral candidiasis. Complete denture wearers are especially susceptible to *C*. *albicans*-associated infections, with denture stomatitis being the most frequent [1]. Denture stomatitis has a multifactorial etiology, with its main causes being poor denture fit, poor denture hygiene and colonization of the denture surface and oral mucosa by *C*. *albicans* [2]. In addition to acting as a physical biofilm reservoir, the presence of the denture base favors greater expression of virulence factors and greater adherence to oral epithelial cells by *C*. *albicans*, resulting in a greater predisposition of oral candidiasis, compared to individuals who do not wear dentures [3].

Complete dentures wearers are mostly elderly individuals, who often present poor manual dexterity for self-care and oral hygiene, and may be at greater risk for systemic diseases [4]. Overnight denture wearing is frequent but dangerous for the elderly, due to the higher risk of aspiration pneumonia [5]. Systemic status can influence the colonization and virulence of *C*. *albicans* as well. *C*. *albicans* isolates from immunosuppressed individuals (e.g., HIV carriers) show intense exoenzymatic activity [6], and even controlled type II diabetes mellitus promotes the expression of candidal proteinase [7]. Those individuals, as well as patients undergoing cancer treatment, present a higher risk of oral and systemic candidiasis [8]. This raises the need for denture base materials to be minimally susceptible to fungal colonization.

The ease of adherence of *C*. *albicans* in polymethylmethacrylate (PMMA) denture base resins can be a source of oral reinfection [9]. Even with the attempts to develop better protocols of hygiene, traditional base materials are susceptible to *C*. *albicans* biofilm formation [10, 11]. It is known that denture base materials vary in terms of adhesion and development of *C*. *albicans*, according to their roughness, hydrophobicity, contact angle and their surface free energy [2, 12–15].

Novel denture base materials should ideally combine a set of properties that may mitigate biofilm formation. Probably the most promising among novel materials are those used for CAD (computer-aided design)/CAM (computer aided-manufacturing). CAM denture base materials have remarkable advantages, including the elimination of some clinical steps, lower costs and reduced clinical/laboratory time [16, 17]. CAM systems can be subtractive (milling) or additive, i.e., 3D printing [16, 17]. The latter has been increasingly used worldwide and employs polymers with more remarkable differences when compared to the conventional heat-polymerized resins. As novel materials, their interaction with oral microorganisms [16], especially in the case of 3D printed resins, is not yet well understood.

Previous studies compared the surface properties of 3D-printed and conventional heat-polymerized denture base resins. Osman et al. demonstrated that the 3D-printed denture base resins resulted in increased candida adhesion and roughest surface than the conventional heat-polymerized and milled denture base resins [18]. Al-Dulaijan et al. showed that the 3D-printed denture base resins (NextDent and ASIGA) exhibited low hardness than the heat-polymerized resin and similar surface roughness, irrespective of the post-curing time and printing orientation [19] However, Al-Dwairi et al. observed that the conventional heat-polymerized denture base resin showed the highest means of surface roughness, Vickers hardness and flexural strength, and the lowest mean of contact angle in comparison to the 3D-printed resins [20].

The 3D printing of denture base resins can use two main techniques: stereolithography (SLA) and digital light processing (DLP). Both types of 3D printers can manufacture denture bases or artificial teeth layer by layer polymerizing light-curing resins [21], with being faster and less expensive [17] than conventional pack-and-press technique. However, the few studies on DLP denture resins do not provide sufficient evidence to understand their interaction with oral biofilms. Even if a study showed no difference between SLA and DLP on the adhesion of *C*. *albicans* to the 3D printed base dentures [22], another study found that a DLP resin can increase early microbial adhesion compared to a conventional heat-polymerized material [23]. Li et al. evaluated different printing-layer thicknesses (25, 50, and 100 μm) and build angles (0°, 45°, and 90°) on surface properties of a denture base resin processed by DLP additive technique [24]. They observed that the adhesion of *C*. *albicans* to the DLP-printed denture surfaces was significantly affected by the printing-layer thickness but not by the build angle; concluding that the layer thickness should be lower than 100μm to avoid the adhesion of *C*. *albicans* [24]. These previous studies emphasizes that different 3D-printed resins and printing parameters may influence on surface properties and *C*. *albicans* adhesion, thus changing clinical performance, and deserve further investigation.

Therefore, this study evaluated adhesion and biofilm formation of *C*. *albicans* on two light-curing resins manufactured by DLP, compared to a heat-polymerized resin. The surface roughness and free energy were also evaluated to explain differences in *Candida* adhesion. The null hypothesis was that would be no difference on formation and metabolism of *C*. *albicans*, roughness and surface free energy, irrespective of the incubation period and type of denture base resin. The results of this study will be relevant in the choice of more favorable parameters for the 3D printing of denture bases with greater longevity and clinical performance, thus promoting oral health to denture wearers.

## Materials and methods

This *in vitro* study compared the NextDent Denture 3D+ (NextDent B.V., Soesterberg, Netherlands) and Cosmos Denture (Yller Digital, Pelotas, RS, Brazil) DLP resins to a control material, i.e., the heat-polymerized acrylic resin Lucitone 550 (Dentsply Ind. e Com. Ltda, Petrópolis, RJ, Brazil). Table 1 shows the composition of the resins [25], printing technology and printing parameters.

**Table 1:** Summary of resins groups.

Summary of resins groups			
**Groups name**	Cosmos Denture (CO)	NextDent Denture 3D+ (NE)	Lucitone 550 (LU)
**Brand name**	Cosmos Denture; Yller Biomateriais AS, Pelotas, RS, Brazil	NextDent Denture 3D+; NextDent B.V., Soesterberg, Netherlands	Lucitone 550; Dentsply Ind. e Com. Ltda, Petrópolis, RJ, Brazil
**Printers**	Flashforge Hunter	Flashforge Hunter	N/A
**LED Wavelenght**	405nm	405nm	
**Printing technology**	LED-based Digital Light Processing (DLP)	LED-based Digital Light Processing (DLP)	N/A
**Composition**	Oligomers, monomers, photoinitiators, stabilizer and pigment	• Ethoxylated bisphenol A dimethacrylate (*>*75%) • 7,7,9(7,9,9)- trimethyl-4,13-dioxo-3,14-dioxa-5,12- diazahexadecane-1,16-diyl bismethacrylate (10–20%) • 2-hydroxyethyl methacrylate (5–10%) • silicon dioxide (1–5%) • Diphenyl (2,4,6-trimethylbenzoyl) phosphine Oxide (1–5%) • Titanium dioxide (*<*0,1%)	Powder: PMMA copolymer, pigments and aesthetic fibers. Liquid: Methyl methacrylate base and inhibitor.
**Orientation**	90°	90°	N/A
**Layer thickness**	50μm	50μm	N/A
**Printed specimens cleaning**	99% isopropyl alcohol	99% isopropyl alcohol	N/A
**Post-polymerization machine**	FormCure; Formlabs, Inc., Somerville, MA, USA	FormCure; Formlabs, Inc., Somerville, MA, USA	N/A
**Post-curing time**	5-min each side	5-min each side	N/A

* N/A: Not Applicable

### Sample size calculation

This study was initially ran with n = 10 specimens/condition, with a power analysis performed subsequently to confirm the adequateness of the sample size. Sample size was determined based on the t-distribution by the Statulator online tool [26]. That analysis was based on the primary variable of the study (log10 CFU/ml), and aimed at detecting a difference of at least 1.0 [27]. Considering the standard deviation obtained by the present study (i.e., 0.4), alpha of 0.05 and power of 0.80, the study needed at least three specimens/condition to reject the null hypothesis.

### Specimen fabrication

**Fig 1:**
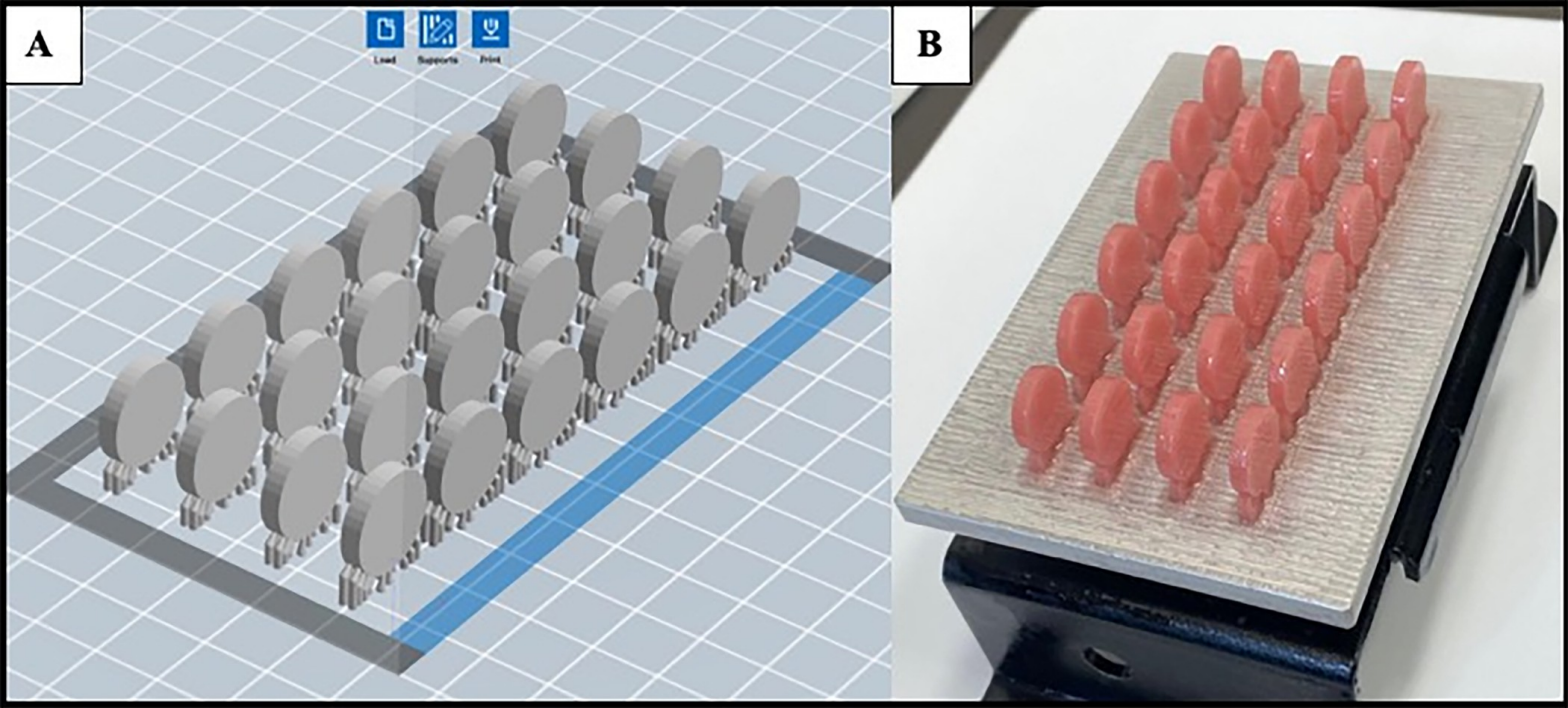
(A) Specimens designed as STL file and (B) printed specimens on the printing platform.

Discs (15mm in diameter and 3mm in thickness) [28] were prepared for each material: NextDent Denture 3D+ (NE, n = 64), Cosmos Denture (CO, n = 64) and Lucitone 550 (LU, n = 64). Group NE and CO specimens were virtually designed as Standard Tessellation Language (STL) files (Adobe Meshmixer v. 3.5; Autodesk Inc, San Rafael, CA, USA, Fig 1A), and loaded in the 3D printer software (FlashDLPrint v. 3.28.0; Flashforge3D Co., Jinhua City, China) for support design and slicing. The printing process was carried out in 90 degrees printing orientation (discs placed with flat surfaces upright, Fig 1B) by a DLP printer (Flashforge Hunter), using ultraviolet light (LED, λ = 405nm, layer thickness of 50 μm) [24, 29, 30]. After printing, discs were removed from the platform and immerged in a container (Form Wash; Formlabs, Inc., Somerville, MA, USA) filled with 99% isopropyl alcohol for 5 min. Subsequently, specimens were taken for post curing at 80°C in UV light (FormCure; Formlabs, Inc., Somerville, MA, USA) for 5 minutes for each sides in a container filled with glycerol [15, 22, 31].

LU specimens (control group) were obtained by the conventional pack-press technique. Metal matrices with cavities with the disc dimensions were included in denture flasks, arranged between glass sheets [32]. The LU acrylic resin was manipulated in the manufacturer’s powder/liquid ratio and inserted into the cavities for pressing. After pressing, the flasks were placed in water bath for 1 ½ hour at 73°C + ½ hour at 100°C in an automatic polymerization tank (Solab Equipamentos para laboratorios Ltda, Piracicaba, SP, Brazil). After polymerization, the flasks were bench cooled, and the specimens were deflasked.

All specimens were finished and polished by the same researcher using the following sequence of wet-dry abrasive papers: 220-, 400- and 600-grit during 10 seconds for each side in a polishing machine (Arotec Ind. E Com. Ltda, Cotia, SP, Brazil) [33, 34]. Before measuring the surface roughness and free energy, the specimens were stored in distilled water at 37°C for 50±2 hours.

### Surface roughness evaluation

The surface roughness in Ra (μm) of all specimens (N = 192; LU n = 64, CO n = 64, NE n = 64) was calculated from three measurements, on a portable digital roughness meter model SJ-400 (Mitutoyo Corporation, Japan) with an accuracy of 0.01 μm and active tip at 0.5 mm/sec speed. From the three measurements obtained of each specimen, an average value of surface roughness in Ra (μm) was calculated for each experimental group.

### Surface free energy evaluation

Surface free energy evaluation used a goniometer (Model 200, Ramé-Hart Instrument co., Netcong, New Jersey, USA) coupled to a computer system that has software (DROPimage Standard, Ramé-Hart Instrument co., Netcong, New Jersey, USA). Distilled water (polar component) and diiodomethane (nonpolar component) were dripped using the same volume and time interval under each specimen (N = 192; LU n = 64, CO n = 64, NE n = 64). Afterwards, the right and left contact angles between the specimen surface and the drops of each liquid were measured. The final contact angle was calculated using the Laplace-Young equation [30]. These values were used to calculate the surface free energy using the OWRK (Owens–Wendt–Rabel–Kaelble) method [35].

### Microbiological assay

#### Inoculum preparation

The microbial strain of *C*. *albicans* used (SC5314) was stored at -80°C and reactivated by the depletion method in plates containing 65g of Sabouraud Dextrose agar + 0.1g Chlorophenicol (SDA) (incubation: 37°C for 48 hours). Then, the pre-inoculum was formed by collecting ten colonies of the reactivated strain, which were transferred to 10 ml of liquid culture medium of Tryptone and Yeast Extract (TYE) supplemented with 1% glucose. After incubation (37° C for 16 hours), the cultures were diluted 1:10 in TYE medium + 1% glucose and the initial optical density (OD) at 540nm was read in a spectrophotometer (CMC Laboratório Ltda., Brazil). The period corresponding to the midlog growth phase was approximately 8 h with OD_540nm_ = 1.07(±0.07) and microbial concentration of 2.31×10^7^(±3.55×10^6^) CFU/mL [36].

#### Saliva collection

Stimulated saliva was collected from eight volunteers for use in forming a salivary glycoprotein film on specimens. This step was approved by the Ethics Committee of the Araraquara School of Dentistry, São Paulo State University **(CAAE: 26410519.0.0000.5416).** Volunteers were selected regardless of sex/gender and age based on the following inclusion criteria: no debilitating systemic disease, fasting for at least and no use of toothpaste or antiseptic mouthwash for two hours before collection; they would be excluded if had taken antibiotics in the last three months.

A total of 400 mL of saliva stimulated by paraffin chewing was collected. The salivary pool was distributed in 50 mL falcon tubes and combined with an adsorption buffer (AB buffer—50 mM KCl, 1 mM KPO_4_, 1 mM CaCl_2_, 1 mM MgCl_2_, in MiliQ water, pH 6.5) in 1:1 proportion. The solution was centrifuged (4°C, 4000RPM, for 10 minutes—Centrifuge 5810R, Eppendorf, Hamburg, Germany) and the supernatant (clarified saliva) was filtered using a 0.22μm polyethersulfone membrane filter (Rapid Flow, Nalgene, Thermo Fisher Scientific, Waltham, Massachusetts, USA). Aliquots of saliva were kept at -80°C until use [36].

#### Sterilization of specimens and planning of microbiological assays

After the surface roughness and surface free energy readings, the specimens were sonicated (1440DA, Odontobrás Equipamentos Médicos e Odontológicas Ltda., Araraquara, SP, Brazil) for 20 minutes in distilled water. Then, both sides of the discs were sterilized for 20 minutes in ultraviolet light in a vertical laminar flow chamber (Model: PA 115, n° 12898, Pachane Indústria e Comércio Ltda., Piracicaba, SP, Brazil) [36].

The specimens were randomly assigned to the microbiological assays, i.e., counting colony forming units per milliliter (CFU/mL; N = 90; LU n = 30, CO n = 30, NE n = 30) and evaluating cell metabolism (XTT assay; N = 90; LU n = 30, CO n = 30, NE n = 30), and inoculation periods. Those two assays were performed in triplicate, on five experiments in different occasions. Analysis by confocal laser scanning microscopy was performed in duplicate only on the fifth occasion (N = 12; LU n = 4, CO n = 4, NE n = 4).

#### Adhesion of *C*. *albicans* and 48-hour biofilm

All specimens underwent salivary film formation before inoculation. The discs were distributed in sterile 12-well plates and immersed in 2 mL of salivary solution and incubated in an orbital shaker (37°C, 75RPM) for 90 minutes.

New sterile 12-well plates received 1 mL of microbial inoculum at the concentration described in topic “Inoculum preparation”, completed with 1 mL of TYE medium + 1% glucose per well. The discs with salivary pellicle were immersed in the wells of the plates and incubated for 90 minutes for the adhesion phase under orbital shaking (37°C, 75RPM).

Half of the discs underwent CFU/mL count and adhesion phase XTT assay immediately after the 90-minute incubation. Specimens were washed once with phosphate-buffered sterile saline (PBS—0.136 M NaCl, 1 mM KH_2_PO_4_, 2 mM KCl, 10 mM Na_2_HPO_4_, pH 7.4) after the 90-minute incubation period and placed on new plates. New 12-well plates were used to prepare the microbial suspension for CFU/mL quantification, each disc was immersed in 2 mL of PBS per well and the surface was scraped individually with a pipette tip for one minute. Finally, serial dilutions of the microbial suspension per specimen disc in each well were plated in SDA culture medium. The other half of the discs were used to assess biofilm formation within 48 hours. It was done immediately after the 90-minute incubation (adhesion phase). The medium was removed and a new sterile TYE culture medium supplemented with 1% sucrose was added to the wells of the same plate, which was kept under orbital shaking (37°C, 75RPM) for 48 hours, with exchange of TYE medium + 1% sucrose in the first 24 hours. After this period, the same washing, scraping and plating protocol was used to quantify the CFU/mL of the adhesion period and the XTT assay was performed [36].

The CFU/mL calculation was obtained by the following formula:

$$\frac{CFU}{mL}=\frac{number\;of\;colonies\times 10^{n}}{q}$$

*n*: absolute value of dilution (1, 2, 3, 4, 5, 6 or 7); chosen for counting that is possible to visualize between 30 to 300 colonies;
*q*: amount, in mL, pipette for each dilution when seeding the plates;
*CFU/mL*: calculate obtained in scientific notation and then arithmetic mean of the triplicate values of each sample. Then, the data obtained for the counts was transformed according to the formula log(CFU+1)/mL.

#### XTT assay

Cell metabolism analysis was performed using the XTT assay. XTT (2,3-bis(2-methoxy-4-nitro-5-sulfophenyl)-5-[(phenylamino)carbonyl]-2H-tetrazolium hydroxide) solution was prepared using PBS, at a concentration of 0.5 g/L, filtered with 0.22μm membrane and stored at -20°C until use. A 10 mM menadione solution was prepared in PA acetone and stored at -20°C until use.

Immediately before the XTT assay, the solution was prepared with 1 μL of menadione added to 10 mL of XTT solution, at a final concentration of 1 μM. After the 48-hour adhesion and biofilm phase, samples that were randomly assigned to the XTT assay were washed once in PBS and placed in sterile 24-well plates. The discs were immersed with 500 μL of the solution in each well, incubated in an orbital shaker (37°C, 75RPM) for 3 hours in a dark environment. Then, 100 μl of the XTT degradation product (supernatant) from each well was pipetted in duplicate to another sterile 96-well plate. The plate was placed in the ELISA reader (Biochrom Ez, Cambourne, UK) to read the absorbance (nm) using the 492 nm filter. Absorbance data represents the optical density of light absorbed at 492nm wavelength. Higher the absorbance, higher the cellular metabolism.

#### Confocal laser scanning microscopy

Cell viability analysis of *C*. *albicans* was performed using confocal laser scanning microscopy (Lsm 800 with Airyscan with GaAsp detector, Carls Zeiss, Germany). After the 48-hour adhesion and biofilm phases, samples randomly assigned to this assay were washed once in PBS and stained with the Live/Dead^TM^ BacLight^TM^ Bacterial Viability Kit (Invitrogen, Thermo Fisher Scientific, Waltham, Massachusetts, USA) containing SYTO-9 and propidium iodide (excitation/emission at 488/400–540 nm and 488/600–700 nm, respectively). For all images, 3% power and pinhole opening of 60μm were used. The resin discs were immersed in 1.5 mL of the dye solution in sterile 12-well plates and incubated in the dark for 45 minutes, as instructed by the manufacturer. After the incubation period, the specimens were washed once in PBS to be observed under confocal laser scanning microscopy. Fluorescence measurement was performed by capturing five images in random fields per disc, totaling ten images per group. Biofilm thickness was determined with z-stack readings at 1 μm [37].

### Statistical analysis

All data collected was verified for normality assumptions (Shapiro-Wilk) and homoscedasticity (Levene). Data analysis of surface roughness (Ra) was performed using the Kruskal-Wallis test followed by the Bonferroni post-hoc test, whereas surface free energy (erg cm^-2^) was analyzed by one-way ANOVA and Tukey HSD. For the microbiological assay, the CFU/mL values were converted into log_10_ for statistical analysis. CFU/mL log_10_ and XTT data were analyzed by two-way ANOVA (factors: type of resin and incubation period). The Tukey HSD post-test was used for the CFU/mL log_10_ and the Bonferroni data for the XTT assay. A descriptive analysis, with confidence intervals, was performed for the fluorescence live/dead and biofilm thickness data obtained by confocal laser scanning microscopy. The SPSS program for Windows (version 15.0; SPSS Inc.) was used for statistical analyses (α = 0.05).

## Results

### Surface roughness and free energy

**Fig 2:**
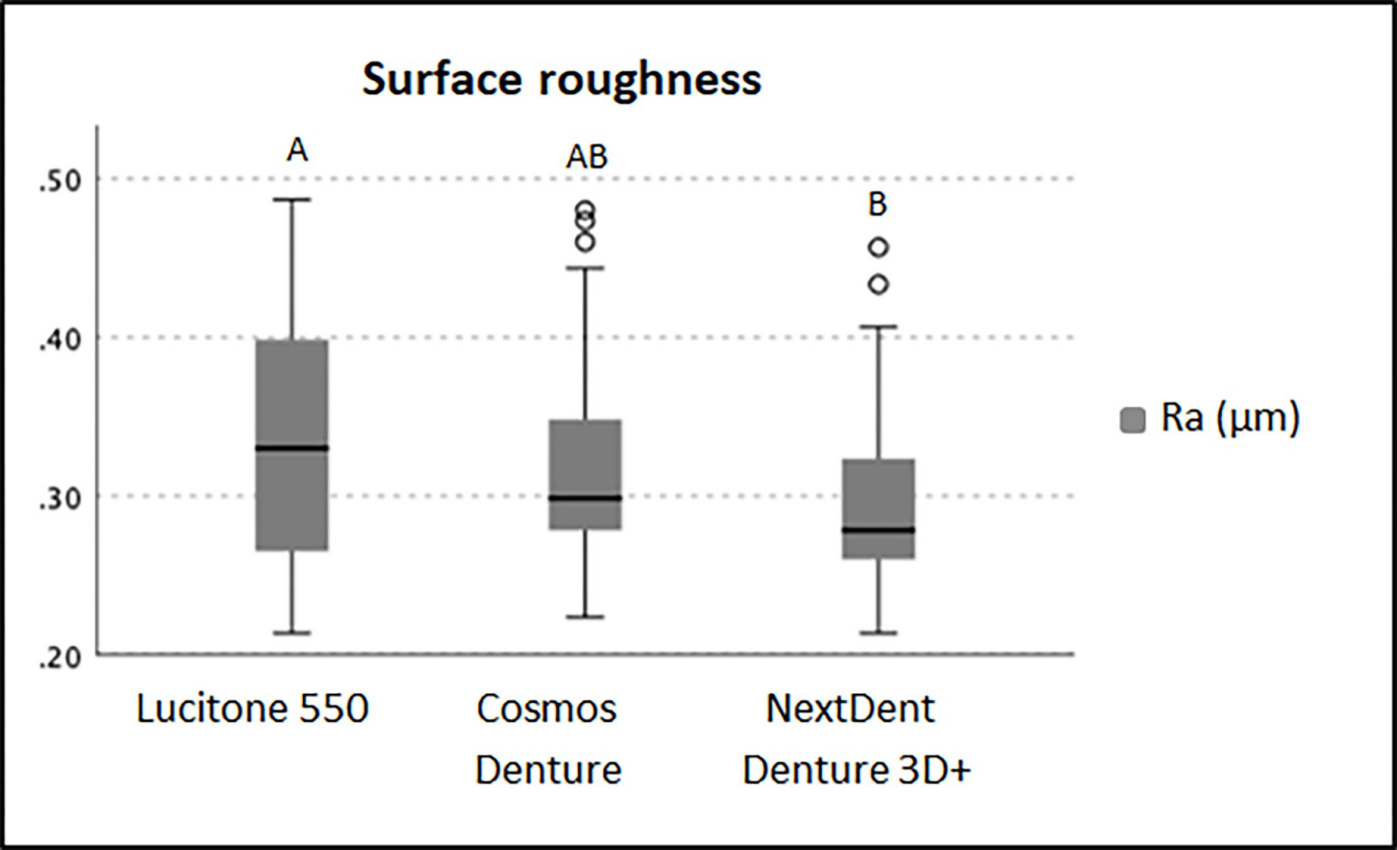
Surface roughness (Ra μm) for the evaluated resins (Kruskal-Wallis and Bonferroni test, p < .05). Similar capital letters indicate statistically significant similarity between groups.

The mean (±SD) Ra for the 3D printed resins was relatively low (NE: 0.295±0.056 μm; CO resin: 0.315±0.058 μm) compared to the control (LU: 0.329±0.07 μm). Differences were significant (Kruskal-Wallis, p = 0.022), with pairwise comparisons (Bonferroni test) showing that the LU resin was significantly rougher than NE (p = 0.024), whereas both LU and NE presented similar roughness to the CO (p = 1.000 and p = 0.129, respectively) (Fig 2).

**Fig 3:**
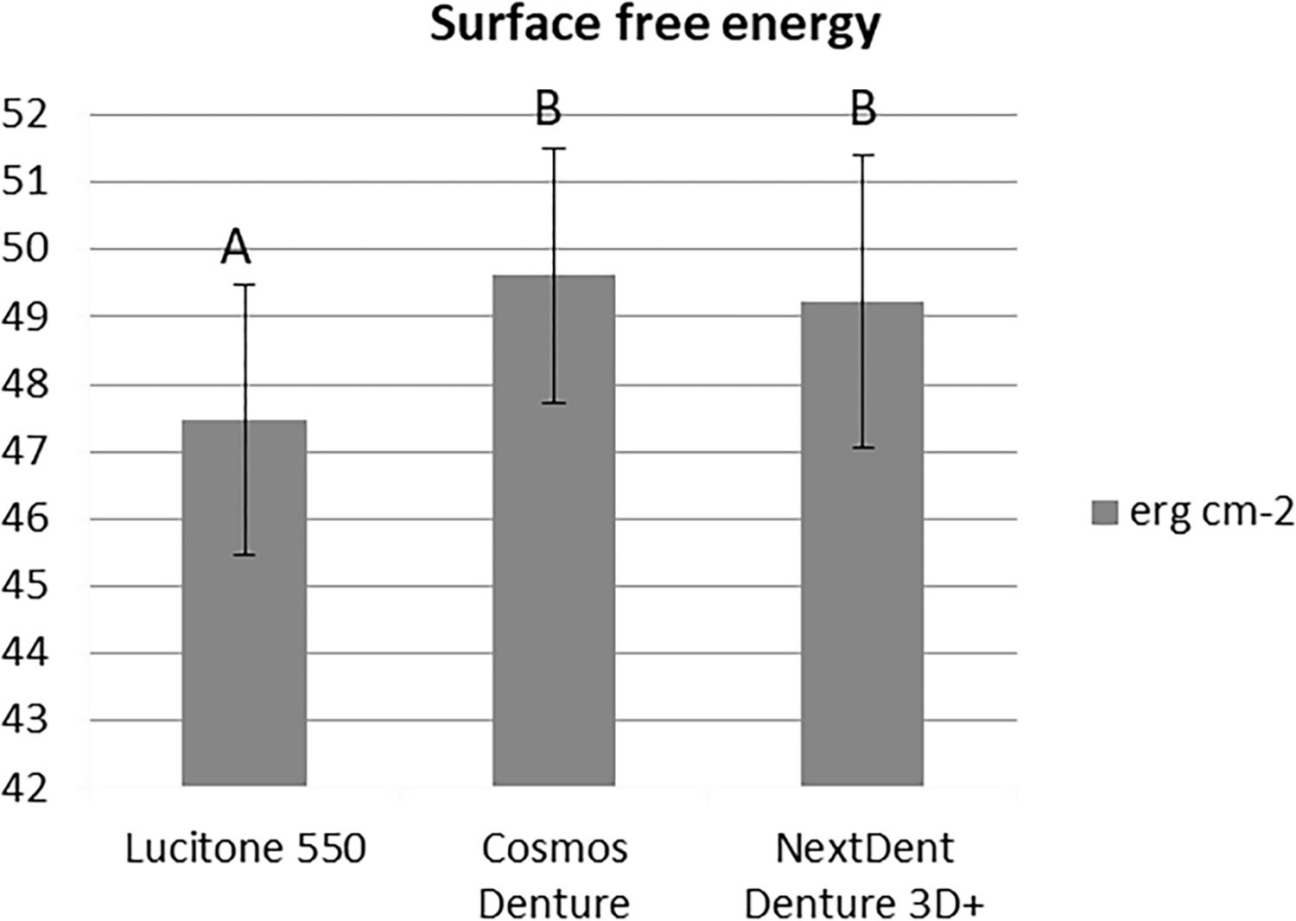
Surface free energy (erg cm^-2^) for the evaluated resins (one-way ANOVA and Tukey HSD, p < .05). Similar capital letters indicate statistically significant similarity between groups.

Both 3D printed resins presented higher surface free energy (CO: 49.61±1.88 erg cm^-2^, NE: 49.23±2.16 erg cm^-2^) than LU (47.47±2.01 erg cm^-2^) (one-way ANOVA, p<0.001, significant). By the Tukey HSD test, differences between LU and both 3D printed resins were significant (p<0.001), but not between CO and NE (p = 0.541) (Fig 3).

### Colony counting

The two-way ANOVA test showed that both the type of resin (p = 0.016) and the incubation period (p<0.001) influenced the quantification of CFU/mL. However, the interaction between the two factors (p = 0.451) was not significant (Table 2).

**Table 2:** Two-way ANOVA of CFU/mL log_10_ counting, according to resin and incubation periods.

Source of variation	SS	Df	MS	F	*P*
**Resin**	0.538	2	0.269	4.965	.016*
**Incubation period**	2.545	1	2.545	46.982	< .001*
**Resin × Incubation period**	0.045	2	0.045	0.824	.451^ns^
**Error**	0.054	24	0.054		

*Significant difference (α = .05)

No significant difference = ns

**Fig 4:**
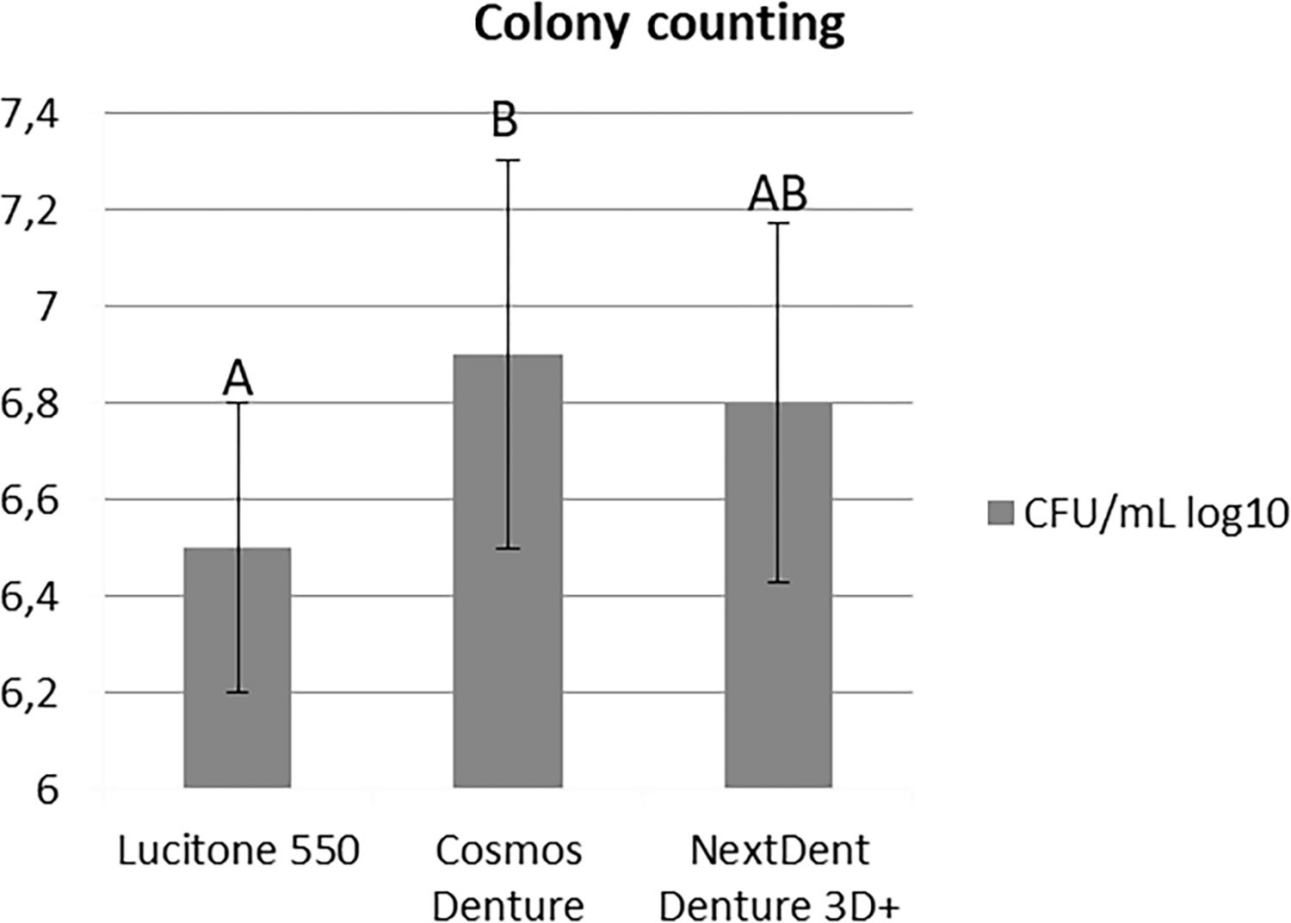
Colony counting (CFU/mL) log_10_ for the evaluated resins (two-way ANOVA and Tukey HSD, p < .05). Similar capital letters indicate statistically significant similarity between groups.

**Fig 5:**
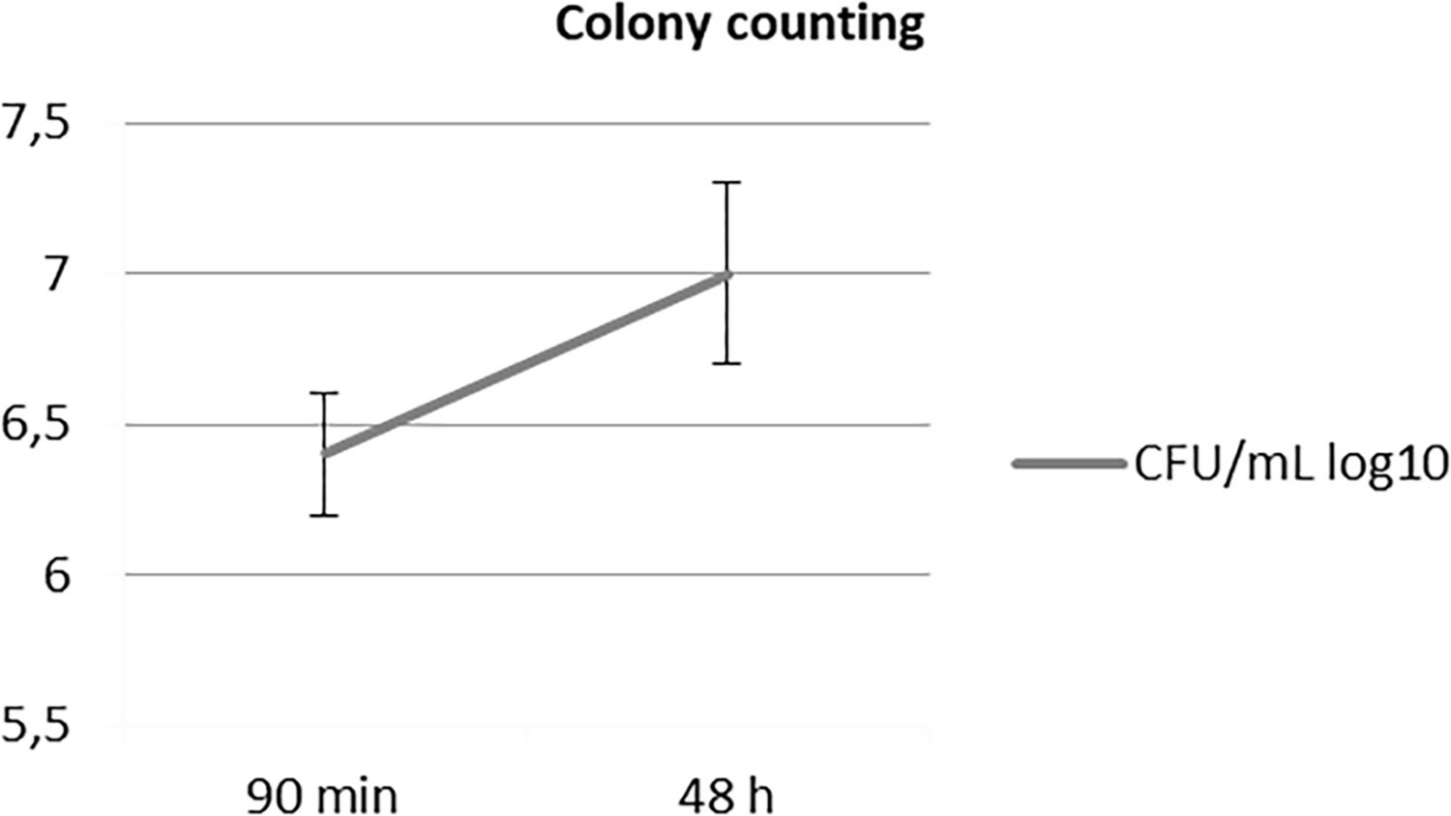
Colony counting (CFU/mL) log_10_ for all the resins, according to the incubation period (two-way ANOVA, p < .05).

The LU acrylic resin of the control group (6.5±0.3) presented lower values in the colony count in relation to the CO resin (6.9±0.4) (p = 0.015), analyzed by the Tukey HSD post-test. However, the LU and CO resins had similar results to the NE resin (6.8±0.37), (p = 0.147 and p = 0.956, respectively) (Fig 4). The adhesion period of 90 minutes (6.4±0.2) showed a lower number of colonies of *C*. *albicans*, in relation to the biofilm of 48 hours (7.0±0.3), irrespective of the resin (Fig 5).

### XTT assay

The two-way ANOVA test only showed the influence of resin type (p<0.001) on cellular metabolism of *C*. *albicans*. However, the incubation period (p = 0.222) and two-factor interaction (p = 0.941) were not significant (Table 3).

**Table 3:** Two-way ANOVA of XTT, according to resin and incubation periods.

Source of variation	SS	Df	MS	F	*P*
**Resin**	0.257	2	0.128	18.590	< .001*
**Incubation period**	0.011	1	0.011	1.574	.222^ns^
**Resin × Incubation period**	0.001	2	0.000	0.061	.941^ns^
**Error**	0.166	24	0.007		

*Significant difference (α = .05)

No significant difference = ns

**Fig 6:**
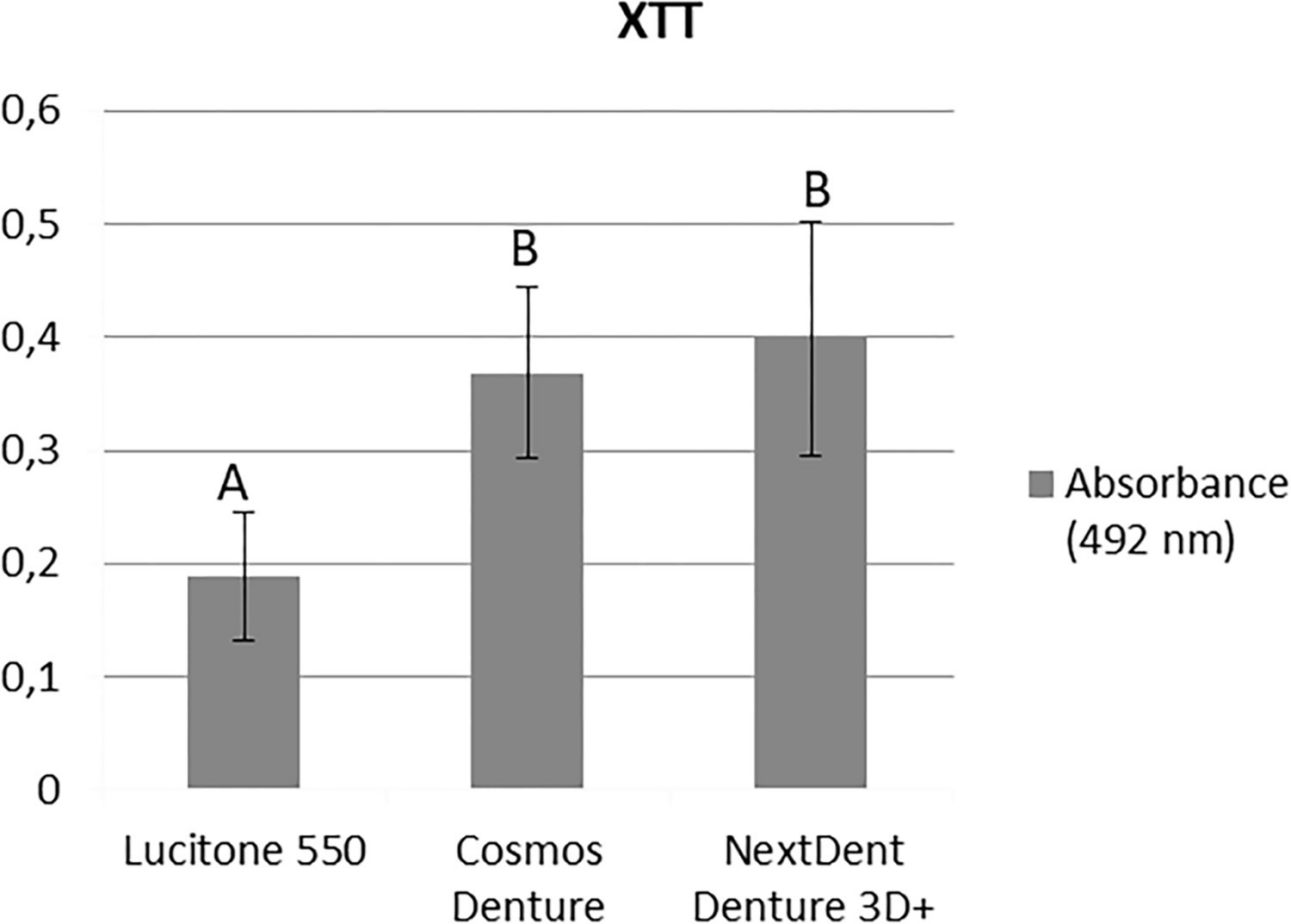
Cell metabolism of *C*. *albicans* (XTT) for the evaluated resins (two-way ANOVA and Bonferroni test, p < .05). Similar capital letters indicate statistically significant similarity between groups.

The 3D printed resins (CO: 0.369±0.076 nm, NE: 0.399±0.104 nm) showed higher cellular metabolism of *C*. *albicans* than LU (0.189±0.056 nm) (Bonferroni post-test, p< 0.001 for both). Both CO and NE had similar results (p = 1.000) (Fig 6).

### Confocal laser scanning microscopy

**Fig 7:**
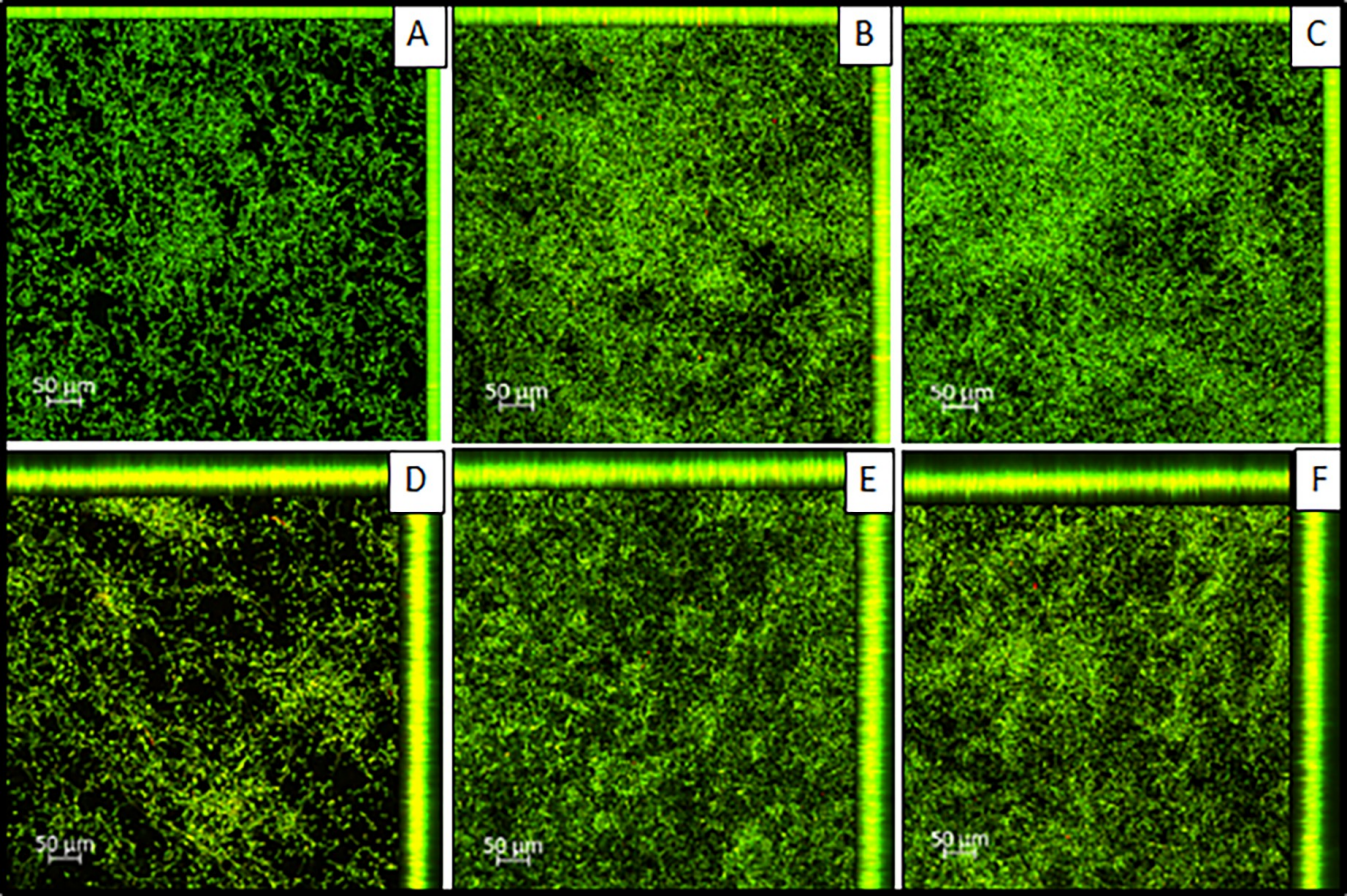
Image captures representing the fluorescence (live/dead) and thickness for 90-min and 48-hour biofilm for LU, CO and NE. A: Lucitone 550 –Adhesion 90min (Z-stack: 11 slices—20μm); B: Cosmos Denture–Adhesion 90 min (Z-stack: 14 slices—26μm); C: NextDent Denture 3D+–Adhesion 90min (Z-stack: 14 slices—26μm); D: Lucitone 550 –Biofilm 48h (Z-stack: 35 slices—68μm); E: Cosmos Denture–Biofilm 48h (Z-stack: 31 slices—60μm); F: NextDent Denture 3D+–Biofilm 48h (Z-stack: 43 slices—84μm). Images information: Live/Dead^TM^ BacLight^TM^ Bacterial Viability Kit; Laser wavelength– 488nm: 3%; Pinhole– 1,88AU / 60μm; Detection wavelength–Live 400-540nm/ Dead 600-700nm; S1–S70 Figs and summary tables (S1–S3 Tables) can be found in the Supporting information section.

The fluorescence quantification data were recorded from the capture of a standard area (411494.395 μm^2^) of the images obtained by group in a 10x objective. Six image captures representing the fluorescence (live/dead) and thickness results for adhesion and 48-hour biofilm periods were selected for the three groups (Table 4) (Fig 7). It was possible to conclude that a greater biofilm thickness and the predominance of live cells was observed in CO resin, followed by NE and finally, the conventional acrylic resin LU (control group), in both periods of 90 minutes of adhesion and biofilm of 48 hours.

**Table 4:** Means, standard deviations and 95% confidence interval of fluorescence (live/dead) and thickness for 90-min and 48-hour biofilm for LU, CO and NE.

Resins		Adhesion			Biofilm 48h		
		Mean / SD	95CI, lower	95CI, upper	Mean / SD	95CI, lower	95CI, upper
**Lucitone 550**	Live	58.1 ±22.7	46.3	70.0	49.3 ±19.9	38.0	60.6
	Dead	24.4 ±12.2	18.0	30.8	26.4 ±10.3	20.6	32.3
	Thickness	20.3 ±2.5	19.0	21.7	48.3 ±4.0	46.1	50.6
**Cosmos Denture**	Live	109.9±33.4	92.4	127.4	79.4 ±32.1	59.5	99.3
	Dead	46.8 ±13.4	39.8	53.9	39.9 ±20.1	27.5	52.4
	Thickness	29.8 ±4.2	27.5	32.0	56.1 ±5.0	53.0	59.2
**NextDent Denture 3D+**	Live	105.7±14.3	97.9	113.4	71.7 ±33.9	52.5	90.9
	Dead	46.2 ±7.4	42.2	50.2	35.6 ±18.3	25.2	46.0
	Thickness	26.3 ±2.8	24.8	27.9	50.3 ±5.3	47.3	53.3

Image captures for fluorescence counting of live/dead microorganisms assumes only a 2D view, which means that the interpretation of these images must consider the biofilm thickness. At 48 h the biofilm thickness was greater for all resins tested, corresponding to almost twice the values of live/dead microorganisms when projecting the values presents in Table 3 in a 3D perspective (considering thickness/depth). Thus, most of the cells remained alive in relation to the adhesion period.

## Discussion

This study investigated surface properties of 3D printed denture base resins and how those resins would interact with *C*. *albicans* compared to a heat-polymerized resin. The null hypothesis was rejected, since the outcomes were influenced by the type of resin and incubation period. The type of resin influenced both colony count and metabolism of *C*. *albicans*, and the incubation period influenced the microbial counting, but did not affect cell metabolism. Moreover, there was a difference in surface roughness and surface free energy among the different resins tested.

The methodologies employed in this study were based on previous investigations. The choice of the printing parameters (printing orientation, layer thickness, wavelength, post-curing time and temperature) may influence the results. According to a previous study, the printing orientation (0, 45 and 90 degrees) did not influence the *C*. *albicans* adhesion on denture base resins [22]. On the other hand, Shim et al. demonstrated that the highest proportion of the *C*.*albicans* was found on the denture bases surfaces printed at an orientation of 0 degrees, followed by 45 and 90 degrees [29]. Thus, the 90-degree printing orientation was more favorable to avoid *C*. *albicans* adhesion [26]. Li et al. [24] evaluated different printing-layer thicknesses (25, 50, and 100 μm) and build angles (0°, 45°, and 90°) on surface properties of a denture base resin processed by DLP additive technique and observed that the adhesion of *C*. *albicans* to the DLP-printed denture surfaces was significantly affected by the printing-layer thickness but not by the build angle. The authors concluded that the layer thickness should be lower than 100μm to avoid the adhesion of *C*. *albicans* [24]. Unkovskiy et al. demonstrated that a 90° build angle provides the best trueness for digital denture fabrication [30]. Altarazi et al. also obtained the best results for mechanical and physical properties of the NextDent 3D-printed resin by printing vertically (90° angle) [38]. These previous studies based for the choice of the 90-degree printing orientation and 50μm printing-layer thickness in our study.

Both tested 3D printed resins had lower roughness than the control material, a traditional PMMA denture base resin. Gad et al. suggested that the low surface roughness of 3D-printed resins may be related to the printing-layer thickness being in the range of 50 μm/layer [39].

The results of this study showed that, despite of the lower roughness, the 3D printed resins, exhibited higher cell metabolism than the control heat-polymerized resin. From these results, it can be suggested that the surface roughness is not the only or the most important factor to determine colonization by *C*. *albicans*. As stated by Gad et al., other factors such as hydrophobicity and contact angle of the resin are also responsible for *C*. *albicans* adhesion [15]. To corroborates with this statement, Shim et al. observed that the higher surface roughness obtained in printed denture resins was not decisive in the amount of *C*. *albicans* adhered, when comparing specimens printed at different angles (0, 45 and 90 degrees) [29].

Another explanation to discuss the lower roughness of 3D printed resins and higher colonization by *C*. *albicans* includes the capability of these materials to absorb different substances. Those materials may absorb salivary and biofilm proteins in different manner when compared to PMMA, as demonstrated for mucin (higher absorption with 3D printed resins) [23]. Lower roughness, however, can be relevant to prevent the harboring of debris and microorganisms by denture bases [2, 40]. This is reinforced by other studies that show no influence of the surface topography/roughness of denture base resins on the adhesion [23], biofilm formation [41] and cell types (hyphae or blastopore) of *C*. *albicans* [42].

Surface free energy of denture base resins was linked to *C*. *albicans* adhesion and biofilm formation in our study. This study endorses that surface free energy can influence microbial adhesion of microorganisms on denture base materials [12]. The 3D-printed resins CO and NE presented the highest surface free energy values in comparison to the control group LU. Despite of the log CFU/ml data showed that the adhesion of the NextDent Denture 3D+ (NE group) was similar to the control group, the 3D-printed specimens exhibited greater cell viability and activity. Thus, it could be hypothesized that the surface free energy may be related to cell viability and activity, but not to cell adherence.

It was stated that surfaces presenting increased free energy contribute to microbial adhesion of *C*. *albicans*, a hydrophobic strain [29, 43]. In this study, the surface free energy of the resins was the main factor to clarify the microbiological findings, showing that the higher surface free energy surfaces, higher the metabolism of *C*. *albicans*, irrespective of the incubation period. Meirowitz et al. demonstrated that the hydrophobicity of denture base resins from different fabrication techniques was moderately associated with microbial cell counts [23]. However, Freitas et al. compared the 3D-printed resin Cosmos Denture with a heat-polymerized resin and showed that the wettability results do not seem to influence the microbiological adhesion on the resin surfaces, but the surface roughness for the authors seemed to make more sense for greater adhesion of *C*. *albicans* biofilm, after 48 hours of incubation [44].

The images obtained from the confocal laser scanning microscopy corroborate with the colony counts and metabolism results obtained in this study, in which a greater amount of live microorganisms and a greater thickness of biofilms was found in the 3D-printed resins, in both incubation periods. Another study presented similar results, since the fabrication of resin samples for denture base using 3D printing increased the amount of colonies in the 4-hour adhesion period of *C*. *albicans* in relation to the conventional technique of heat-polymerized [23]. On the other hand, Fiore et al. verified greater adhesion of *C*. *albicans* in 90 minutes on the heat-polymerized PMMA resin in comparison to the 3D-printed by SLA technique and milled denture base resins, but all resins had similar microbial adhesion after 16 hours of incubation [45]. Osman et al. observed higher *Candida* adhesion after 24-hour incubation on printed resins compared to conventional heat-polymerized unpolished and even after polishing the specimens [18]. The authors attributed these results to the gradual joining between the printed layers resulting in increased porosities and deep grooves observed in the surface structure by optical analysis using field emission scanning electron microscope [18].

The need for greater knowledge about the composition of resins used in digital additive manufacturing is a limitation of the present study. Restricted patents by manufacturing companies do not allow for a more accurate exploration of the results found. Furthermore, the use of different brands of printers and post-curing units (time and temperature) from the same resin manufacturer may also be a limitation of this study, as stated by a recent systematic review [46]. However, it was possible to verify that the resins used in 3D printing have higher surface free energy compared to conventional acrylic resins, making them more avid to colonize microorganisms and greater cellular metabolism of *C*. *albicans*.

The simulation of the oral environment is needed to obtain more consistent outcomes and conclusions. The results of this study cannot state whether the formation of the salivary film influenced the surface free energy of the resin samples, which could also represent a limitation of the study. Future studies should be carried out to evaluate the morphology of *C*. *albicans* adhered on the surfaces of 3D-printed resins since it could influence the virulence and adherence of this microorganism, representing a primordial factor in the development of oral pathologies [14, 42, 47, 48]. Moreover, the predictability and applicability of these materials in clinical routine rely on clinical trials to assess their long-term behavior.

This *in vitro* study presents relevant findings about the microbiological behavior of 3D-printed denture base resins concerning the adherence and metabolism of *C*. *albicans*, the main etiological factor to the development of denture stomatitis, the most prevalent oral pathology among denture wearers. Therefore, these results could be useful to support future *in vivo* approaches, and finally the safe indication of these resins to fabricate denture bases ensuring the health of their users.

### Conclusions

Both 3D printed resins (Cosmos Denture and NextDent Denture 3D+) are slightly more prone to colonization by *C*. *albicans* than a conventional heat-polymerized denture base resin. The surface free energy of the resins was an important factor behind those findings, since the 3D-printed resins presented the highest surface free energy values in comparison to the heat-polymerized resin and exhibited greater cell viability and activity. On the other hand, differences in their surface roughness were not associated with microbial colonization.

## Supporting information

S1 Fig First image capture of Lucitone 550 specimen in confocal with 90min adhesion period. (TIFF)

S2 Fig Second image capture of Lucitone 550 specimen in confocal with 90min adhesion period. (TIFF)

S3 Fig Third image capture of Lucitone 550 specimen in confocal with 90min adhesion period. (TIFF)

S4 Fig Fourth image capture of Lucitone 550 specimen in confocal with 90min adhesion period. (TIFF)

S5 Fig Fifth image capture of Lucitone 550 specimen in confocal with 90min adhesion period. (TIFF)

S6 Fig Sixth image capture of Lucitone 550 specimen in confocal with 90min adhesion period. (TIFF)

S7 Fig Seventh image capture of Lucitone 550 specimen in confocal with 90min adhesion period. (TIFF)

S8 Fig Eighth image capture of Lucitone 550 specimen in confocal with 90min adhesion period. (TIFF)

S9 Fig Ninth image capture of Lucitone 550 specimen in confocal with 90min adhesion period. (TIFF)

S10 Fig Tenth image capture of Lucitone 550 specimen in confocal with 90min adhesion period. (TIFF)

S11 Fig Eleventh image capture of Lucitone 550 specimen in confocal with 90min adhesion period. (TIFF)

S12 Fig Twelfth image capture of Lucitone 550 specimen in confocal with 90min adhesion period. (TIFF)

S13 Fig First image capture of Lucitone 550 specimen in confocal with 48-hour biofilm period. (TIFF)

S14 Fig Second image capture of Lucitone 550 specimen in confocal with 48-hour biofilm period. (TIFF)

S15 Fig Third image capture of Lucitone 550 specimen in confocal with 48-hour biofilm period. (TIFF)

S16 Fig Fourth image capture of Lucitone 550 specimen in confocal with 48-hour biofilm period. (TIFF)

S17 Fig Fifth image capture of Lucitone 550 specimen in confocal with 48-hour biofilm period. (TIFF)

S18 Fig Sixth image capture of Lucitone 550 specimen in confocal with 48-hour biofilm period. (TIFF)

S19 Fig Seventh image capture of Lucitone 550 specimen in confocal with 48-hour biofilm period. (TIFF)

S20 Fig Eighth image capture of Lucitone 550 specimen in confocal with 48-hour biofilm period. (TIFF)

S21 Fig Ninth image capture of Lucitone 550 specimen in confocal with 48-hour biofilm period. (TIFF)

S22 Fig Tenth image capture of Lucitone 550 specimen in confocal with 48-hour biofilm period. (TIFF)

S23 Fig Eleventh image capture of Lucitone 550 specimen in confocal with 48-hour biofilm period. (TIFF)

S24 Fig Twelfth image capture of Lucitone 550 specimen in confocal with 48-hour biofilm period. (TIFF)

S25 Fig First image capture of Cosmos Denture specimen in confocal with 90min adhesion period. (TIFF)

S26 Fig Second image capture of Cosmos Denture specimen in confocal with 90min adhesion period. (TIFF)

S27 Fig Third image capture of Cosmos Denture specimen in confocal with 90min adhesion period. (TIFF)

S28 Fig Fourth image capture of Cosmos Denture specimen in confocal with 90min adhesion period. (TIFF)

S29 Fig Fifth image capture of Cosmos Denture specimen in confocal with 90min adhesion period. (TIFF)

S30 Fig Sixth image capture of Cosmos Denture specimen in confocal with 90min adhesion period. (TIFF)

S31 Fig Seventh image capture of Cosmos Denture specimen in confocal with 90min adhesion period. (TIFF)

S32 Fig Eighth image capture of Cosmos Denture specimen in confocal with 90min adhesion period. (TIFF)

S33 Fig Ninth image capture of Cosmos Denture specimen in confocal with 90min adhesion period. (TIFF)

S34 Fig Tenth image capture of Cosmos Denture specimen in confocal with 90min adhesion period. (TIFF)

S35 Fig Eleventh image capture of Cosmos Denture specimen in confocal with 90min adhesion period. (TIFF)

S36 Fig Twelfth image capture of Cosmos Denture specimen in confocal with 90min adhesion period. (TIFF)

S37 Fig First image capture of Cosmos Denture specimen in confocal with 48-hour biofilm period. (TIFF)

S38 Fig Second image capture of Cosmos Denture specimen in confocal with 48-hour biofilm period. (TIFF)

S39 Fig Third image capture of Cosmos Denture specimen in confocal with 48-hour biofilm period. (TIFF)

S40 Fig Fourth image capture of Cosmos Denture specimen in confocal with 48-hour biofilm period. (TIFF)

S41 Fig Fifth image capture of Cosmos Denture specimen in confocal with 48-hour biofilm period. (TIFF)

S42 Fig Sixth image capture of Cosmos Denture specimen in confocal with 48-hour biofilm period. (TIFF)

S43 Fig Seventh image capture of Cosmos Denture specimen in confocal with 48-hour biofilm period. (TIFF)

S44 Fig Eighth image capture of Cosmos Denture specimen in confocal with 48-hour biofilm period. (TIFF)

S45 Fig Ninth image capture of Cosmos Denture specimen in confocal with 48-hour biofilm period. (TIFF)

S46 Fig Tenth image capture of Cosmos Denture specimen in confocal with 48-hour biofilm period. (TIFF)

S47 Fig First image capture of NextDent Denture 3D+ specimen in confocal with 90min adhesion period. (TIFF)

S48 Fig Second image capture of NextDent Denture 3D+ specimen in confocal with 90min adhesion period. (TIFF)

S49 Fig Third image capture of NextDent Denture 3D+ specimen in confocal with 90min adhesion period. (TIFF)

S50 Fig Fourth image capture of NextDent Denture 3D+ specimen in confocal with 90min adhesion period. (TIFF)

S51 Fig Fifth image capture of NextDent Denture 3D+ specimen in confocal with 90min adhesion period. (TIFF)

S52 Fig Sixth image capture of NextDent Denture 3D+ specimen in confocal with 90min adhesion period. (TIFF)

S53 Fig Seventh image capture of NextDent Denture 3D+ specimen in confocal with 90min adhesion period. (TIFF)

S54 Fig Eighth image capture of NextDent Denture 3D+ specimen in confocal with 90min adhesion period. (TIFF)

S55 Fig Ninth image capture of NextDent Denture 3D+ specimen in confocal with 90min adhesion period. (TIFF)

S56 Fig Tenth image capture of NextDent Denture 3D+ specimen in confocal with 90min adhesion period. (TIFF)

S57 Fig Eleventh image capture of NextDent Denture 3D+ specimen in confocal with 90min adhesion period. (TIFF)

S58 Fig Twelfth image capture of NextDent Denture 3D+ specimen in confocal with 90min adhesion period. (TIFF)

S59 Fig First image capture of NextDent Denture 3D+ specimen in confocal with 48-hour biofilm period. (TIFF)

S60 Fig Second image capture of NextDent Denture 3D+ specimen in confocal with 48-hour biofilm period. (TIFF)

S61 Fig Third image capture of NextDent Denture 3D+ specimen in confocal with 48-hour biofilm period. (TIFF)

S62 Fig Fourth image capture of NextDent Denture 3D+ specimen in confocal with 48-hour biofilm period. (TIFF)

S63 Fig Fifth image capture of NextDent Denture 3D+ specimen in confocal with 48-hour biofilm period. (TIFF)

S64 Fig Sixth image capture of NextDent Denture 3D+ specimen in confocal with 48-hour biofilm period. (TIFF)

S65 Fig Seventh image capture of NextDent Denture 3D+ specimen in confocal with 48-hour biofilm period. (TIFF)

S66 Fig Eighth image capture of NextDent Denture 3D+ specimen in confocal with 48-hour biofilm period. (TIFF)

S67 Fig Ninth image capture of NextDent Denture 3D+ specimen in confocal with 48-hour biofilm period. (TIFF)

S68 Fig Tenth image capture of NextDent Denture 3D+ specimen in confocal with 48-hour biofilm period. (TIFF)

S69 Fig Eleventh image capture of NextDent Denture 3D+ specimen in confocal with 48-hour biofilm period. (TIFF)

S70 Fig Twelfth image capture of NextDent Denture 3D+ specimen in confocal with 48-hour biofilm period. (TIFF)

S1 Table Descriptive values of the supplementary figures corresponding to the adhesion and biofilm periods of Lucitone 550 resin. * Image chosen to represent the group in Fig 6.
(DOCX)

S2 Table Descriptive values of the supplementary figures corresponding to the adhesion and biofilm periods of Cosmos Denture resin. * Image chosen to represent the group in Fig 6.
(DOCX)

S3 Table Descriptive values of the supplementary figures corresponding to the adhesion and biofilm periods of NextDent Denture 3D+ resin. * Image chosen to represent the group in Fig 6.
(DOCX)
