# Supplementary figures and images for: Microbial adhesion and biofilm formation by Candida albicans on 3D-printed denture base resins
Source: PLoS One. 2023 Oct 4;18(10):e0292430. doi: 10.1371/journal.pone.0292430 (PMC10550158; doi:10.1371/journal.pone.0292430)

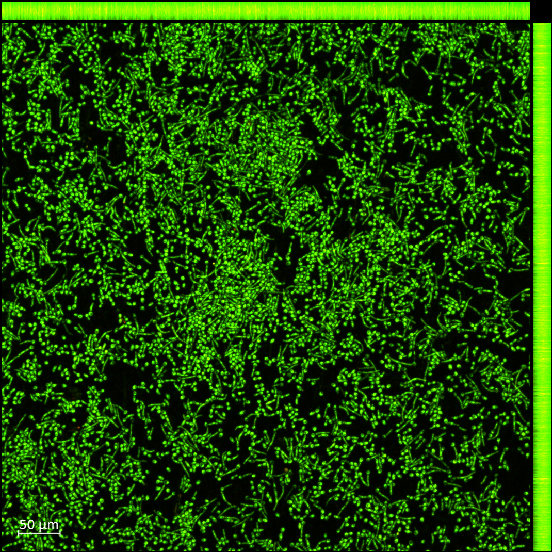

Supplement: S1 Fig — (TIFF) [file pone.0292430.s001.tiff]

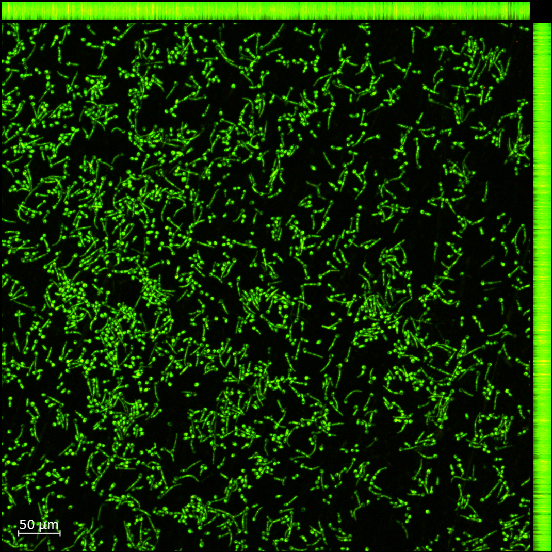

Supplement: S2 Fig — (TIFF) [file pone.0292430.s002.tiff]

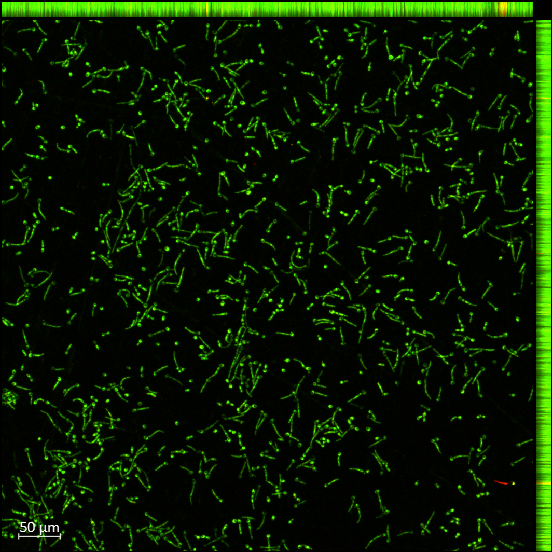

Supplement: S3 Fig — (TIFF) [file pone.0292430.s003.tiff]

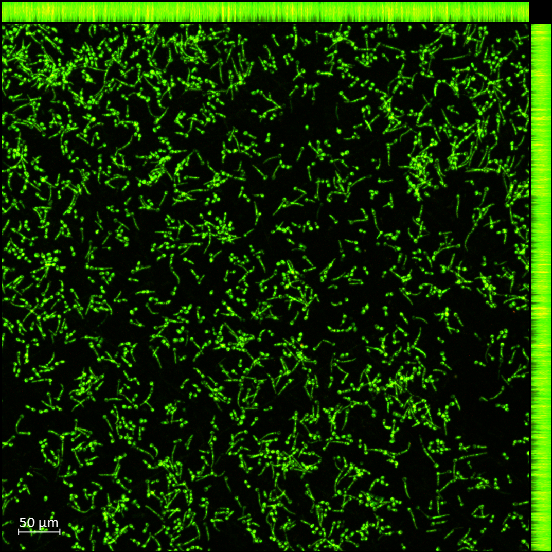

Supplement: S4 Fig — (TIFF) [file pone.0292430.s004.tiff]

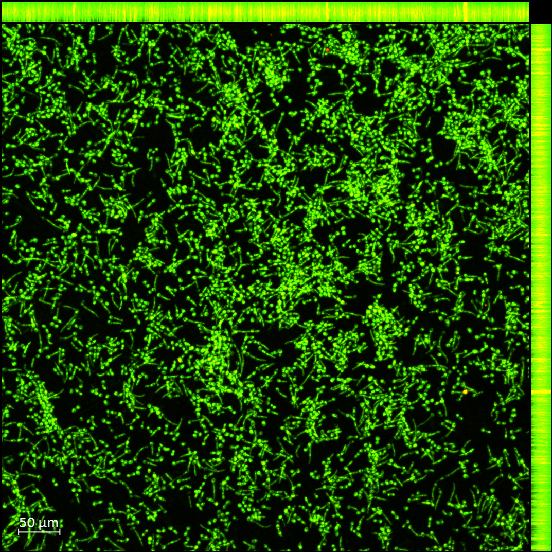

Supplement: S5 Fig — (TIFF) [file pone.0292430.s005.tiff]

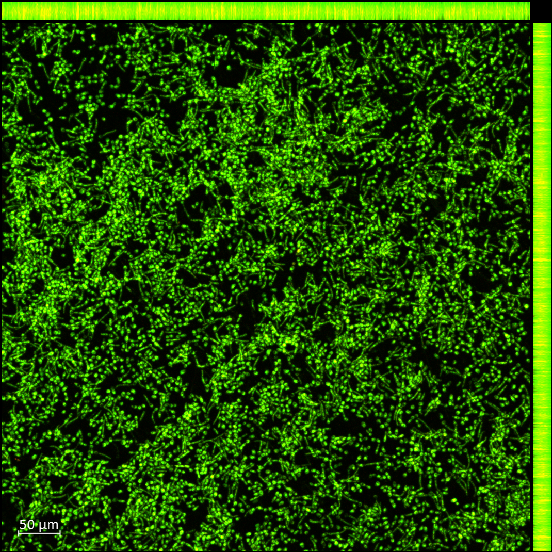

Supplement: S6 Fig — (TIFF) [file pone.0292430.s006.tiff]

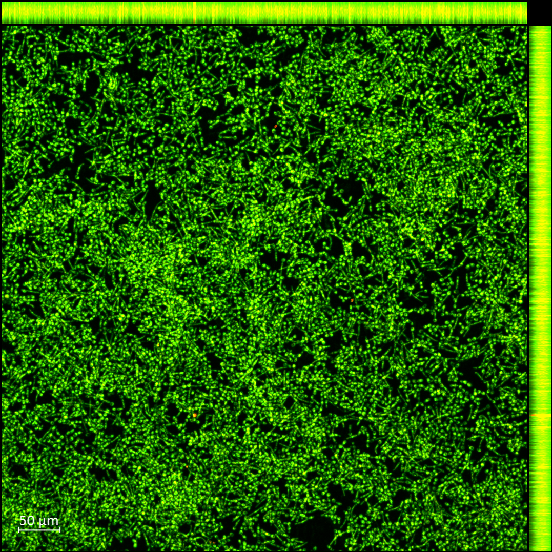

Supplement: S7 Fig — (TIFF) [file pone.0292430.s007.tiff]

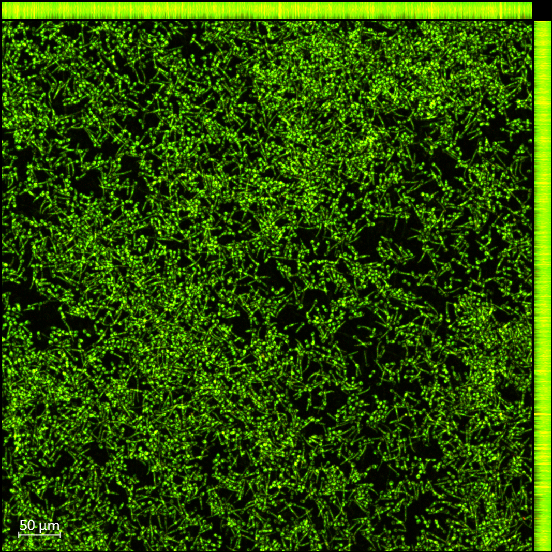

Supplement: S8 Fig — (TIFF) [file pone.0292430.s008.tiff]

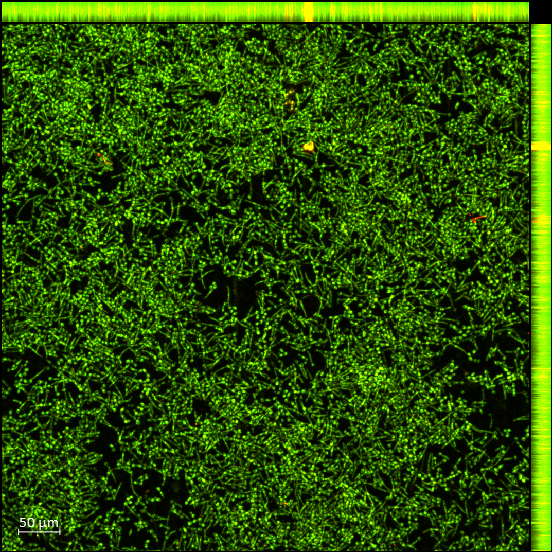

Supplement: S9 Fig — (TIFF) [file pone.0292430.s009.tiff]

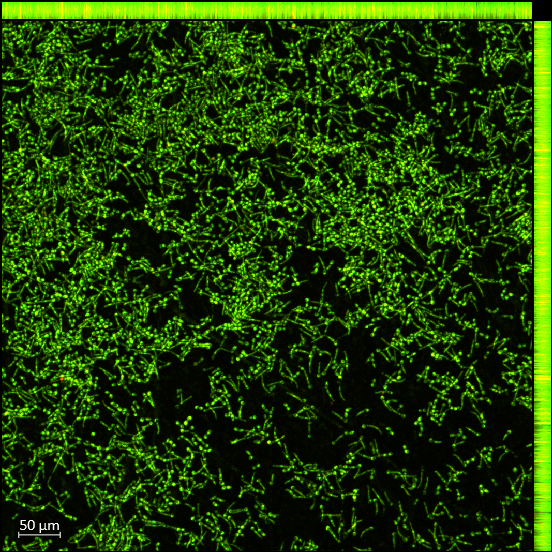

Supplement: S10 Fig — (TIFF) [file pone.0292430.s010.tiff]

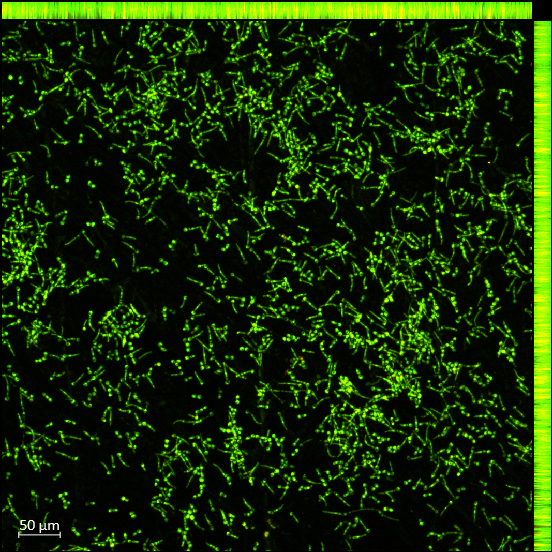

Supplement: S11 Fig — (TIFF) [file pone.0292430.s011.tiff]

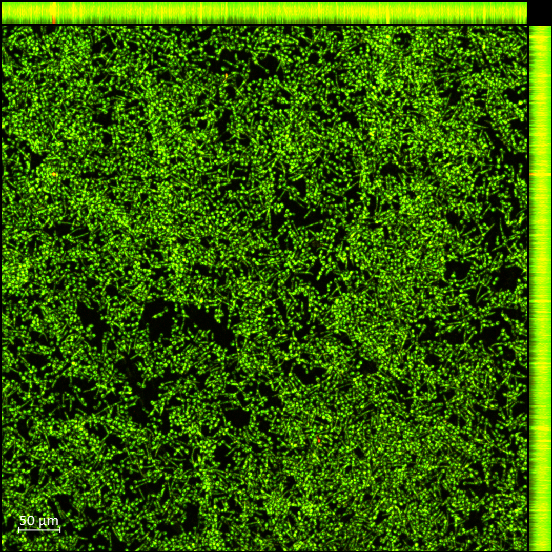

Supplement: S12 Fig — (TIFF) [file pone.0292430.s012.tiff]

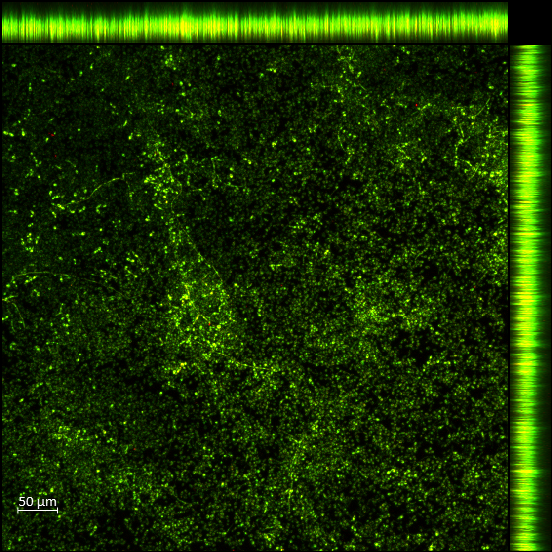

Supplement: S13 Fig — (TIFF) [file pone.0292430.s013.tiff]

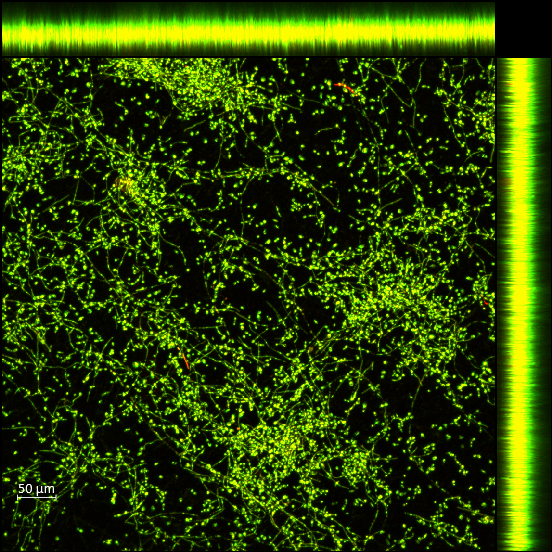

Supplement: S14 Fig — (TIFF) [file pone.0292430.s014.tiff]

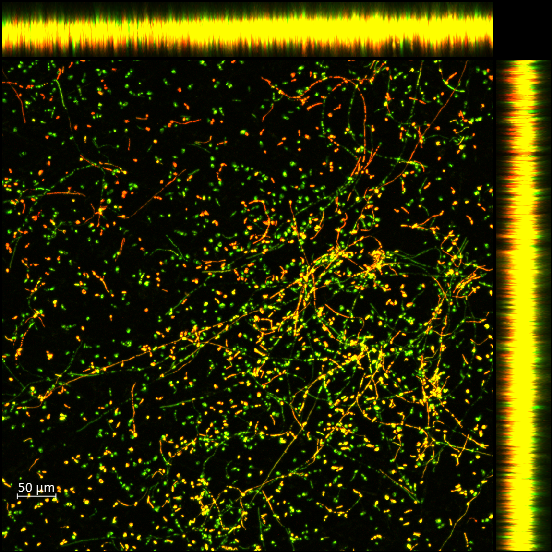

Supplement: S15 Fig — (TIFF) [file pone.0292430.s015.tiff]

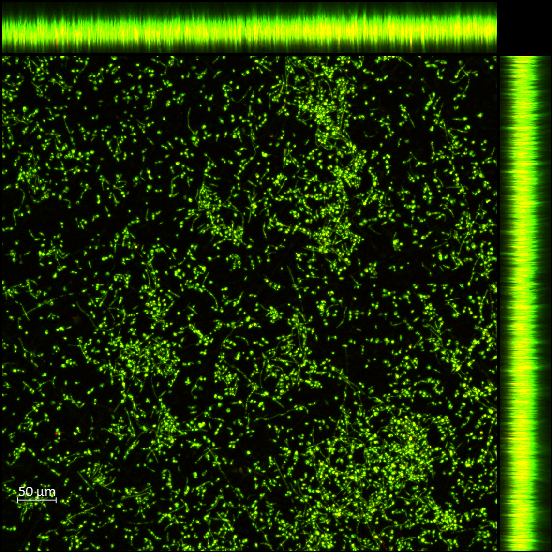

Supplement: S16 Fig — (TIFF) [file pone.0292430.s016.tiff]

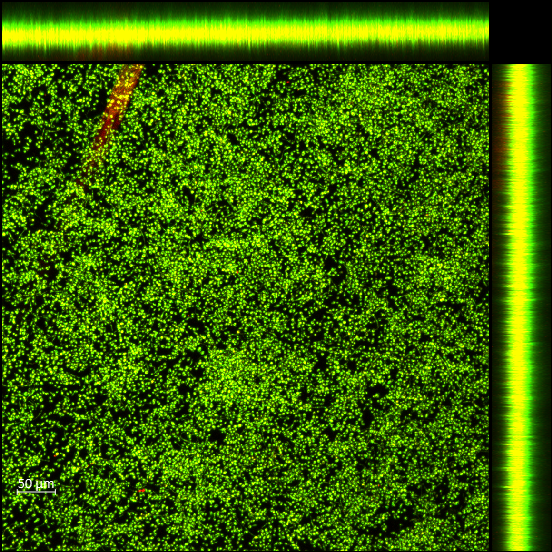

Supplement: S17 Fig — (TIFF) [file pone.0292430.s017.tiff]

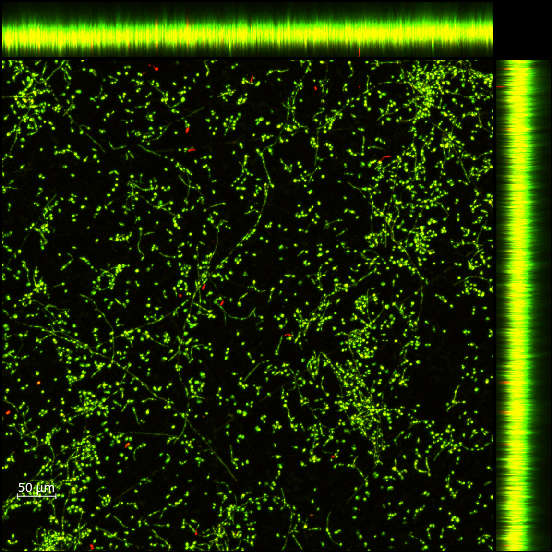

Supplement: S18 Fig — (TIFF) [file pone.0292430.s018.tiff]

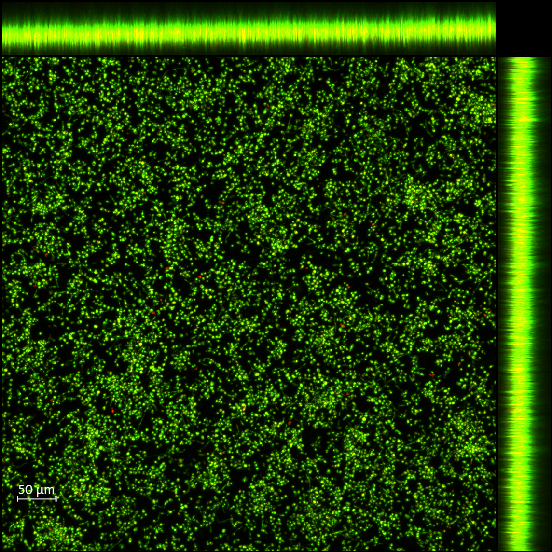

Supplement: S19 Fig — (TIFF) [file pone.0292430.s019.tiff]

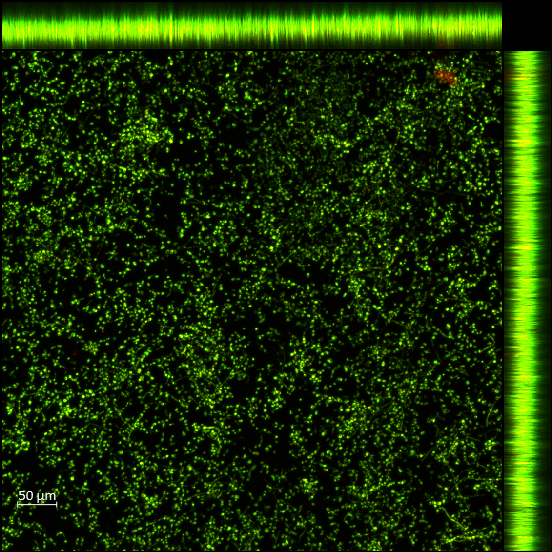

Supplement: S20 Fig — (TIFF) [file pone.0292430.s020.tiff]

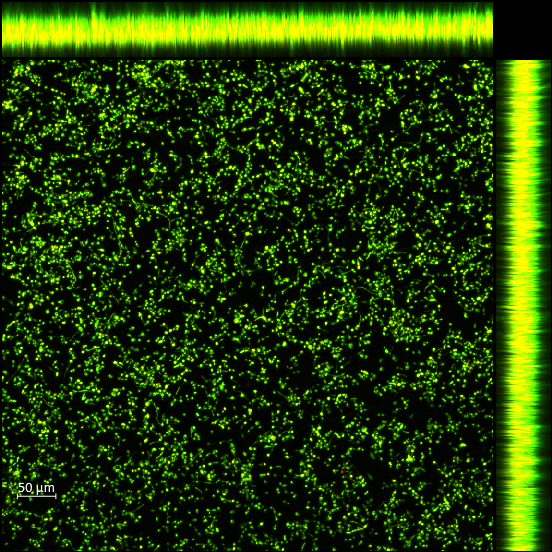

Supplement: S21 Fig — (TIFF) [file pone.0292430.s021.tiff]

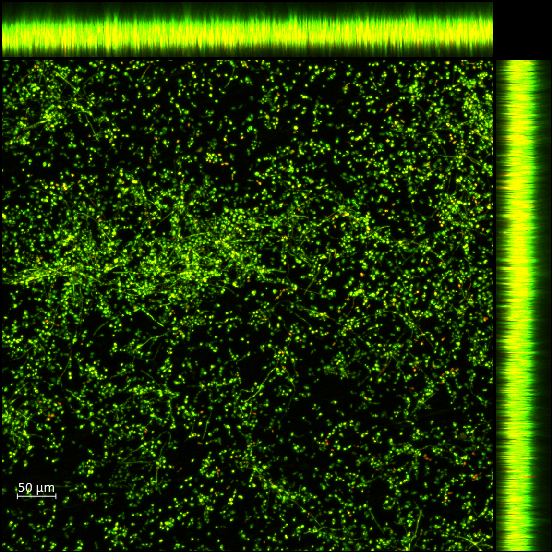

Supplement: S22 Fig — (TIFF) [file pone.0292430.s022.tiff]

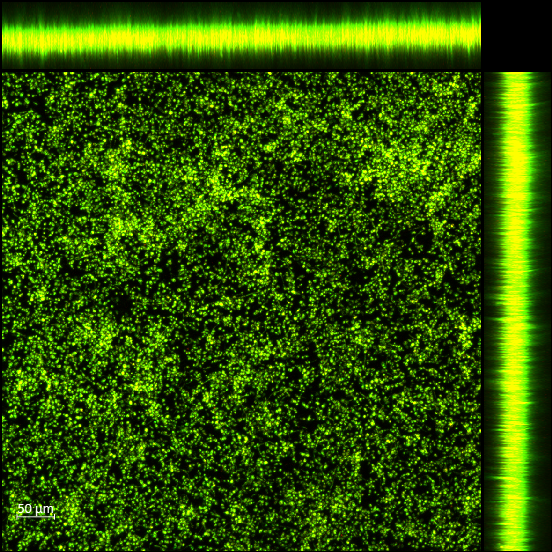

Supplement: S23 Fig — (TIFF) [file pone.0292430.s023.tiff]

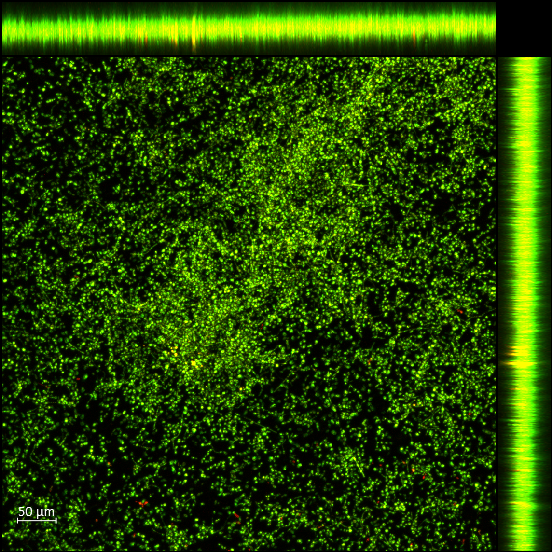

Supplement: S24 Fig — (TIFF) [file pone.0292430.s024.tiff]

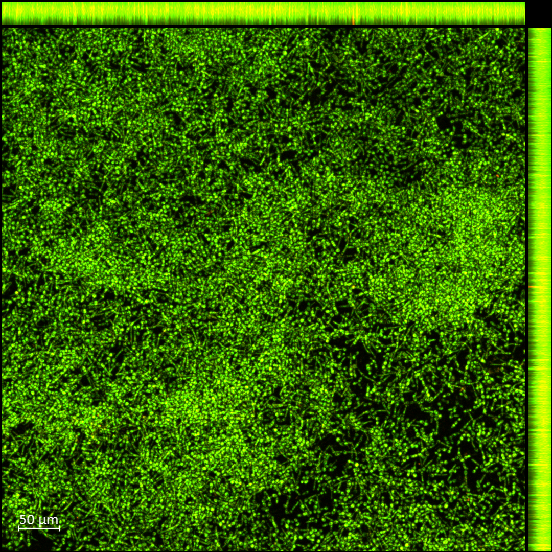

Supplement: S25 Fig — (TIFF) [file pone.0292430.s025.tiff]

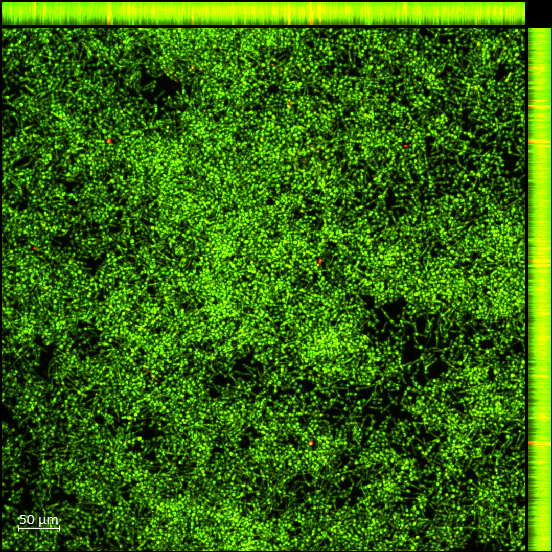

Supplement: S26 Fig — (TIFF) [file pone.0292430.s026.tiff]

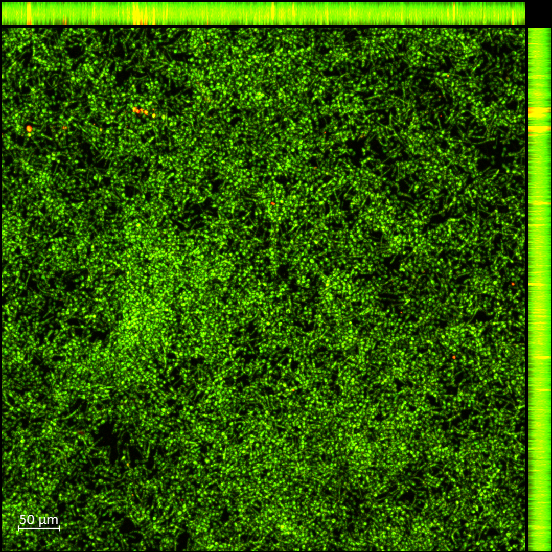

Supplement: S27 Fig — (TIFF) [file pone.0292430.s027.tiff]

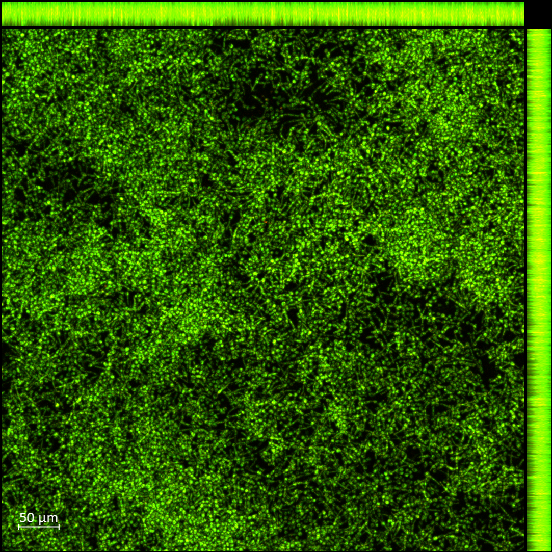

Supplement: S28 Fig — (TIFF) [file pone.0292430.s028.tiff]

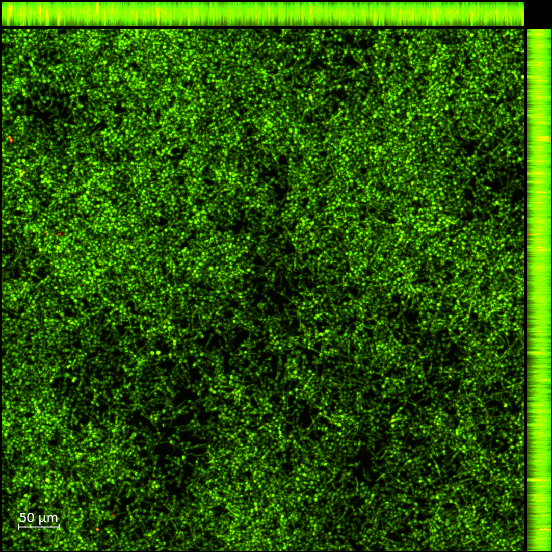

Supplement: S29 Fig — (TIFF) [file pone.0292430.s029.tiff]

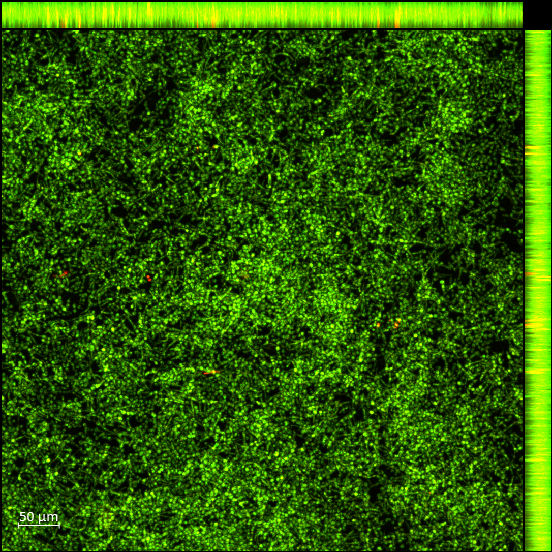

Supplement: S30 Fig — (TIFF) [file pone.0292430.s030.tiff]

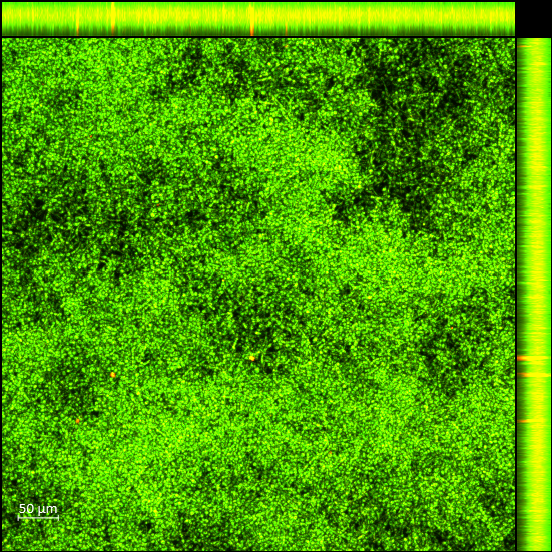

Supplement: S31 Fig — (TIFF) [file pone.0292430.s031.tiff]

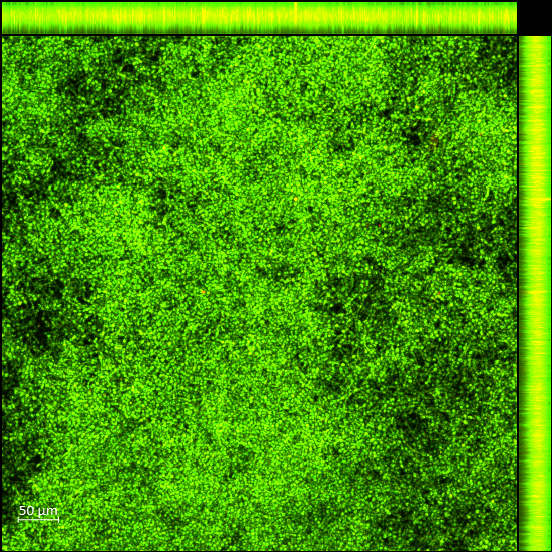

Supplement: S32 Fig — (TIFF) [file pone.0292430.s032.tiff]

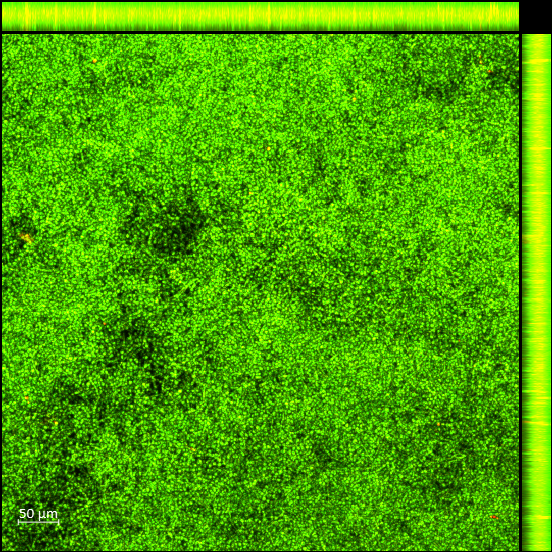

Supplement: S33 Fig — (TIFF) [file pone.0292430.s033.tiff]

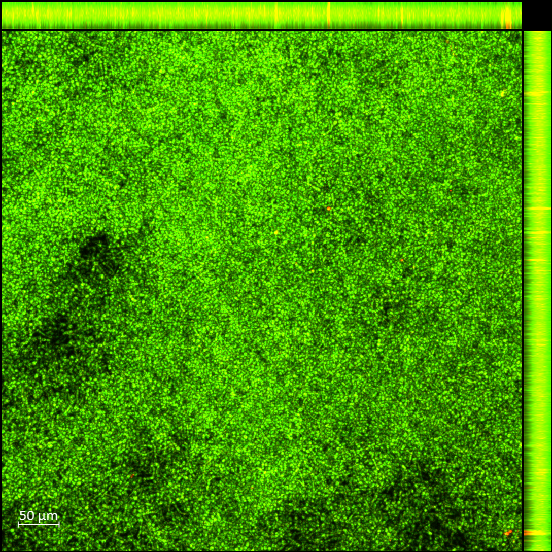

Supplement: S34 Fig — (TIFF) [file pone.0292430.s034.tiff]

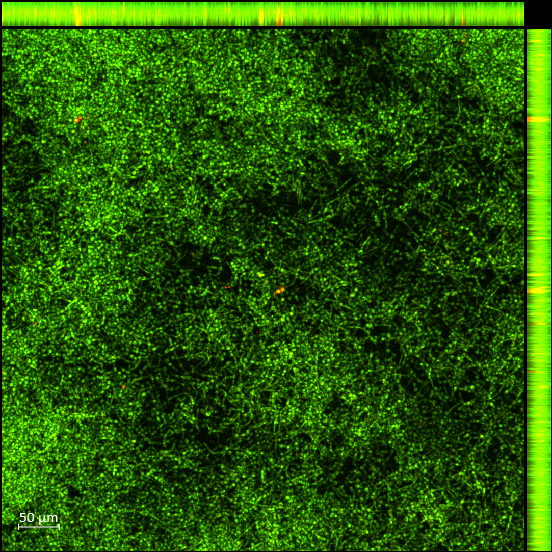

Supplement: S35 Fig — (TIFF) [file pone.0292430.s035.tiff]

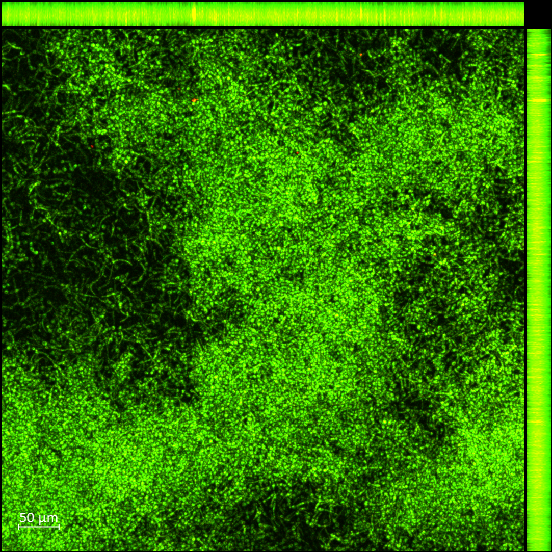

Supplement: S36 Fig — (TIFF) [file pone.0292430.s036.tiff]

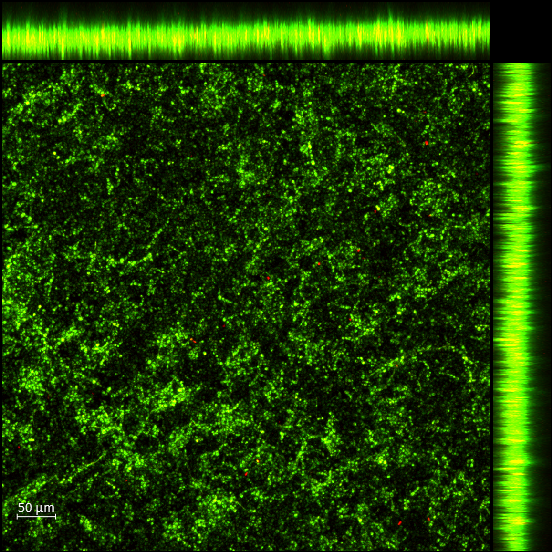

Supplement: S37 Fig — (TIFF) [file pone.0292430.s037.tiff]

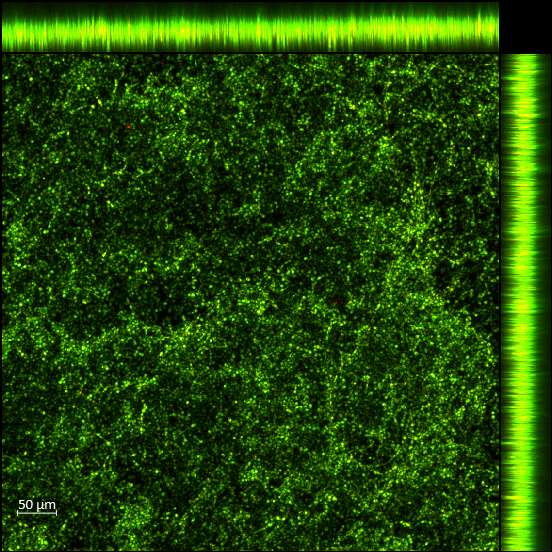

Supplement: S38 Fig — (TIFF) [file pone.0292430.s038.tiff]

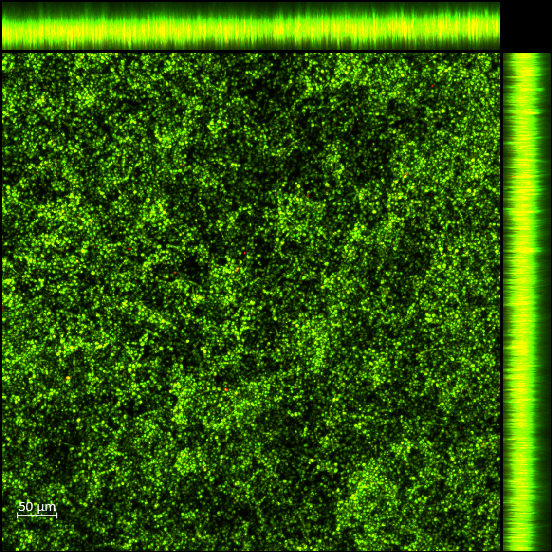

Supplement: S39 Fig — (TIFF) [file pone.0292430.s039.tiff]

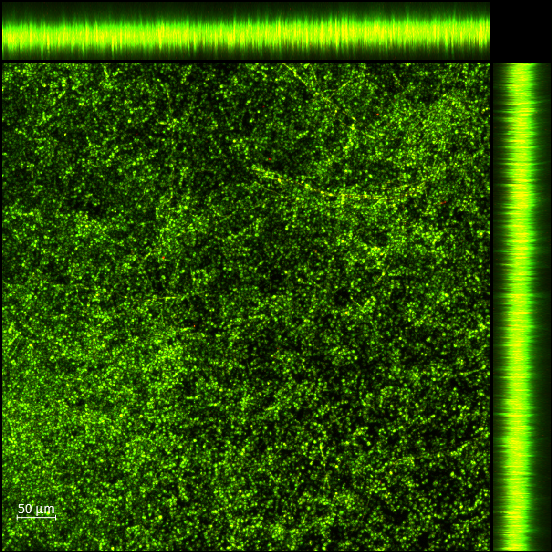

Supplement: S40 Fig — (TIFF) [file pone.0292430.s040.tiff]

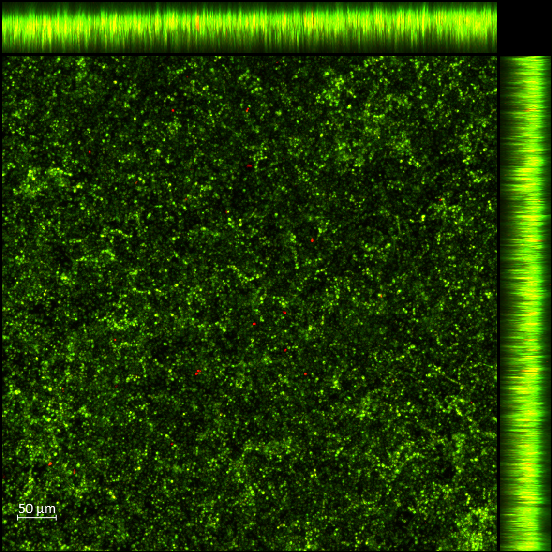

Supplement: S41 Fig — (TIFF) [file pone.0292430.s041.tiff]

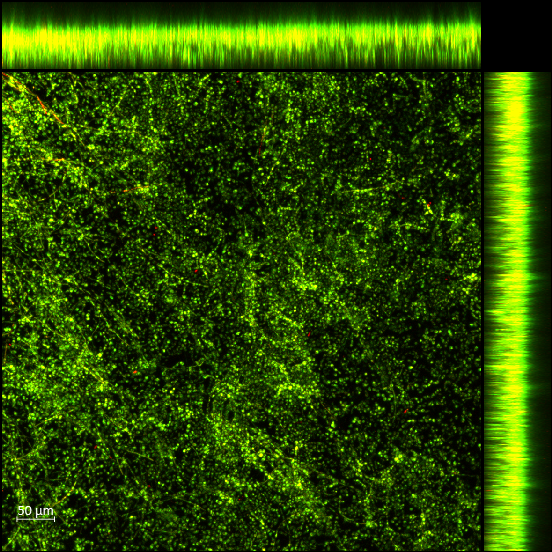

Supplement: S42 Fig — (TIFF) [file pone.0292430.s042.tiff]

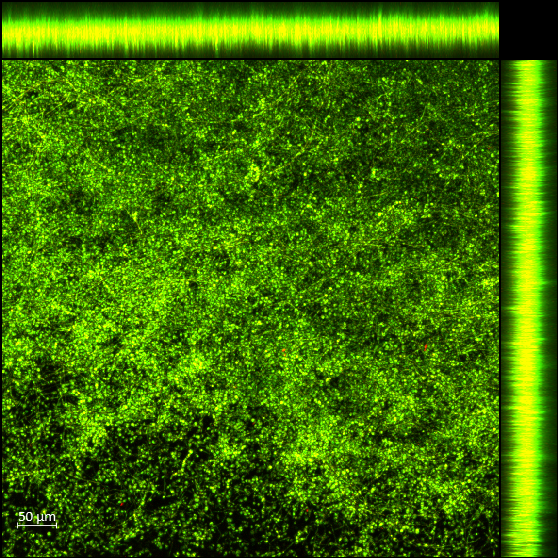

Supplement: S43 Fig — (TIFF) [file pone.0292430.s043.tiff]

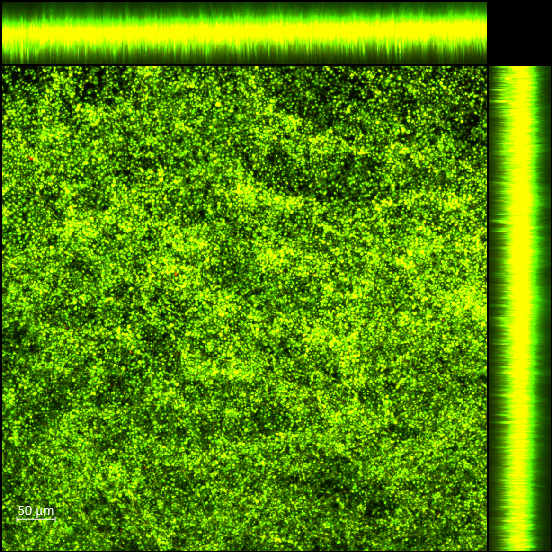

Supplement: S44 Fig — (TIFF) [file pone.0292430.s044.tiff]

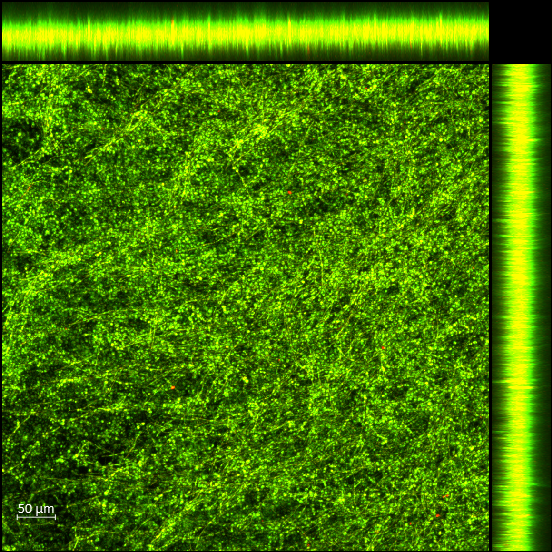

Supplement: S45 Fig — (TIFF) [file pone.0292430.s045.tiff]

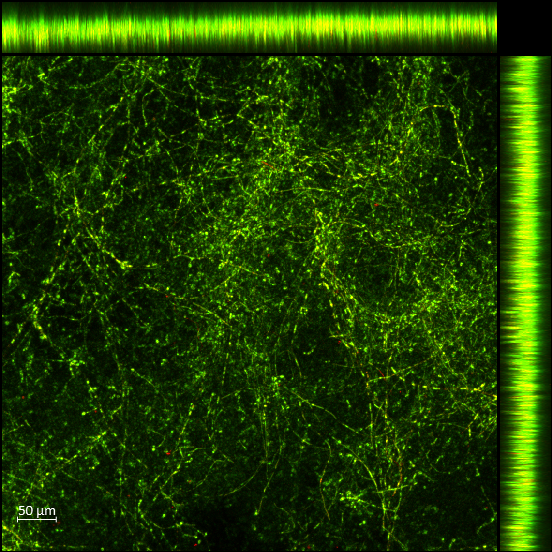

Supplement: S46 Fig — (TIFF) [file pone.0292430.s046.tiff]

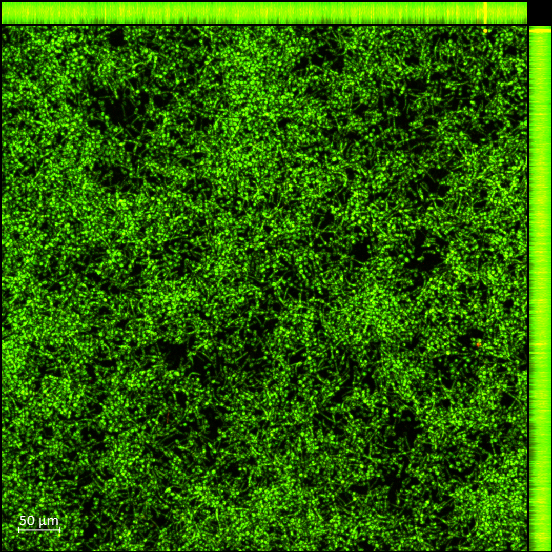

Supplement: S47 Fig — (TIFF) [file pone.0292430.s047.tiff]

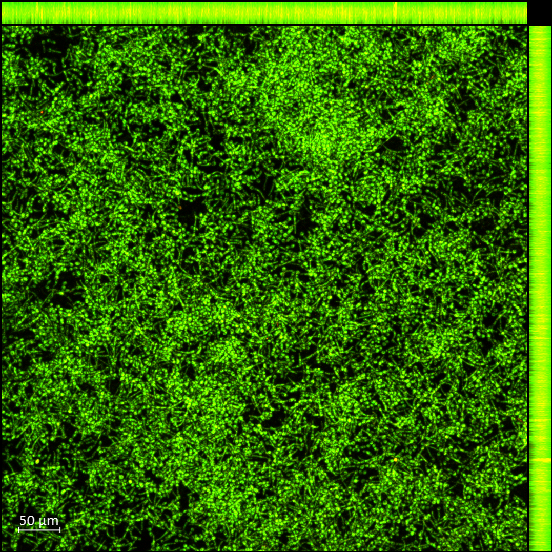

Supplement: S48 Fig — (TIFF) [file pone.0292430.s048.tiff]

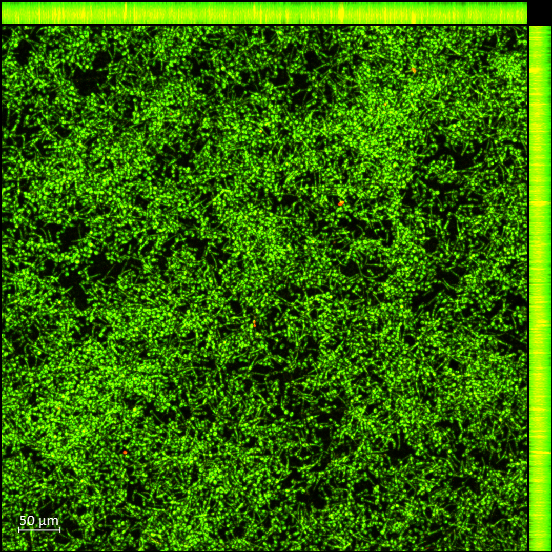

Supplement: S49 Fig — (TIFF) [file pone.0292430.s049.tiff]

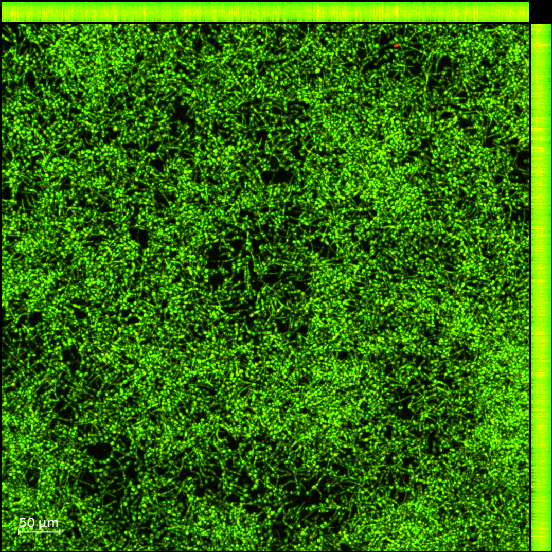

Supplement: S50 Fig — (TIFF) [file pone.0292430.s050.tiff]

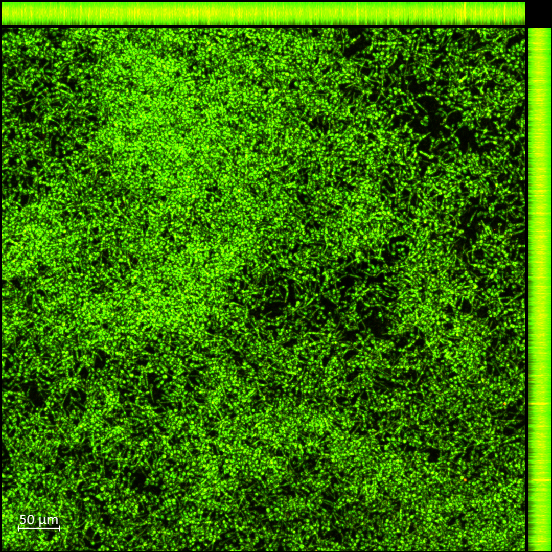

Supplement: S51 Fig — (TIFF) [file pone.0292430.s051.tiff]

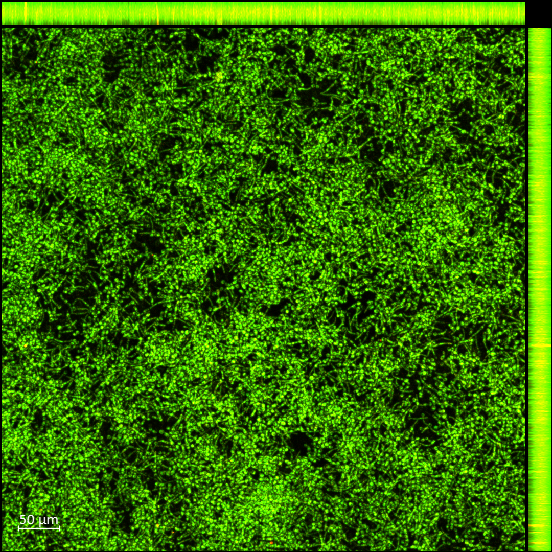

Supplement: S52 Fig — (TIFF) [file pone.0292430.s052.tiff]

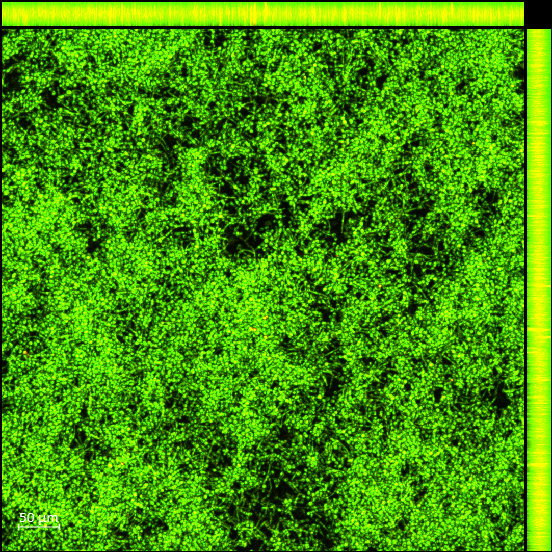

Supplement: S53 Fig — (TIFF) [file pone.0292430.s053.tiff]

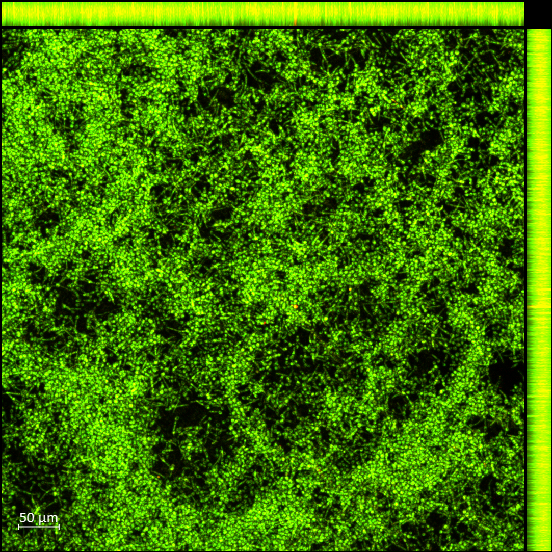

Supplement: S54 Fig — (TIFF) [file pone.0292430.s054.tiff]

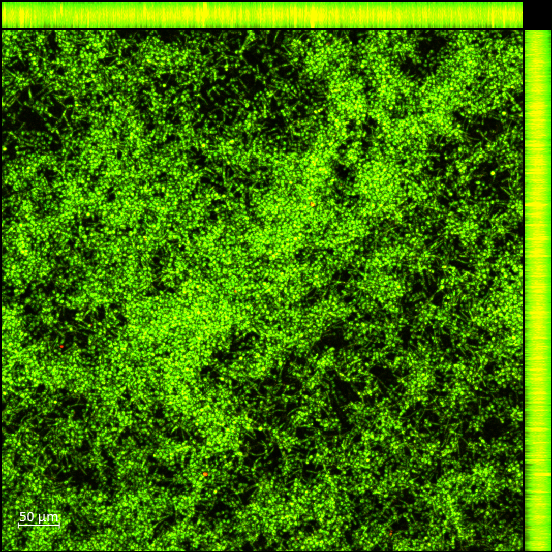

Supplement: S55 Fig — (TIFF) [file pone.0292430.s055.tiff]

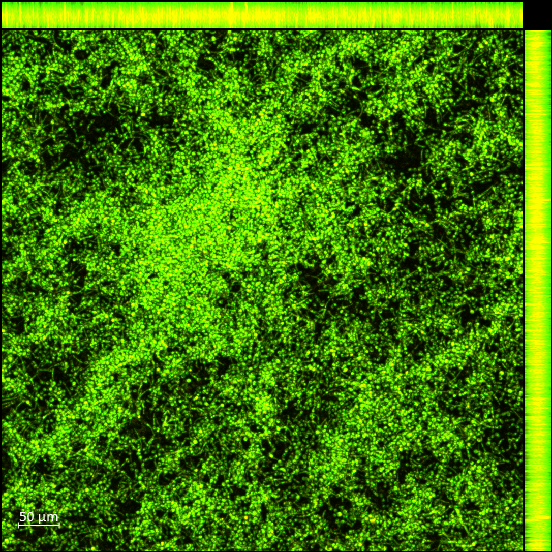

Supplement: S56 Fig — (TIFF) [file pone.0292430.s056.tiff]

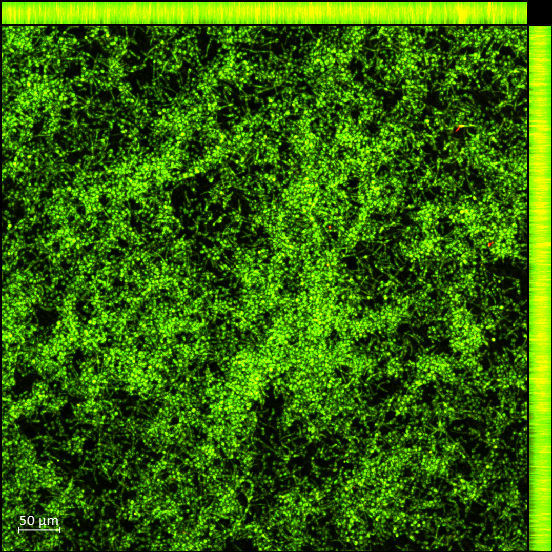

Supplement: S57 Fig — (TIFF) [file pone.0292430.s057.tiff]

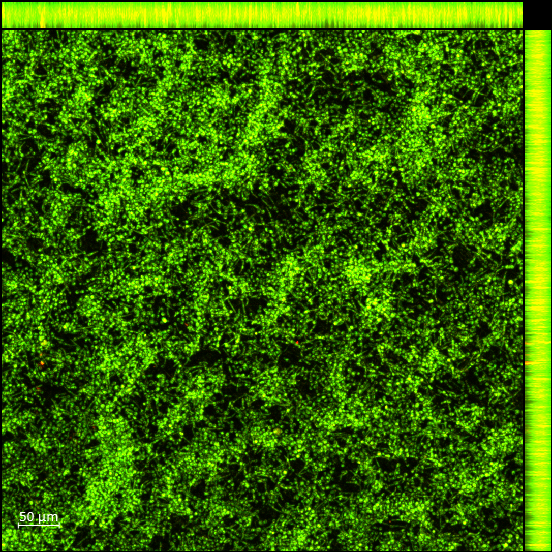

Supplement: S58 Fig — (TIFF) [file pone.0292430.s058.tiff]

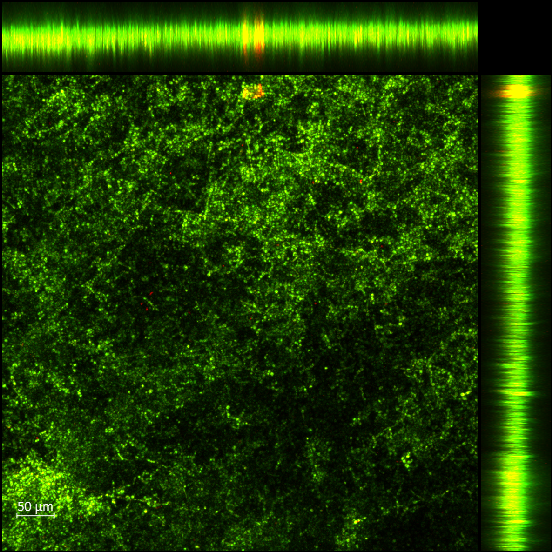

Supplement: S59 Fig — (TIFF) [file pone.0292430.s059.tiff]

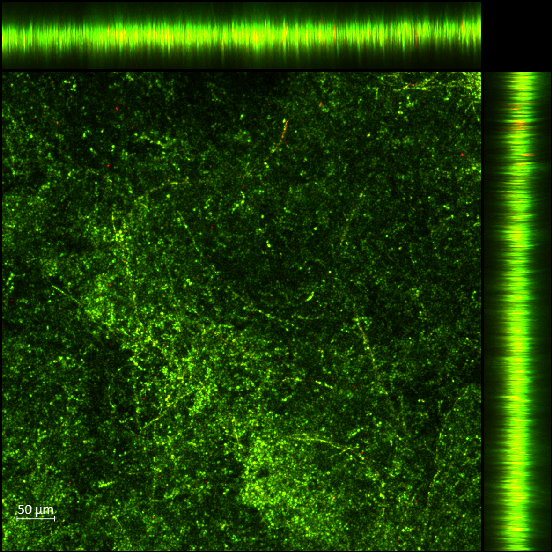

Supplement: S60 Fig — (TIFF) [file pone.0292430.s060.tiff]

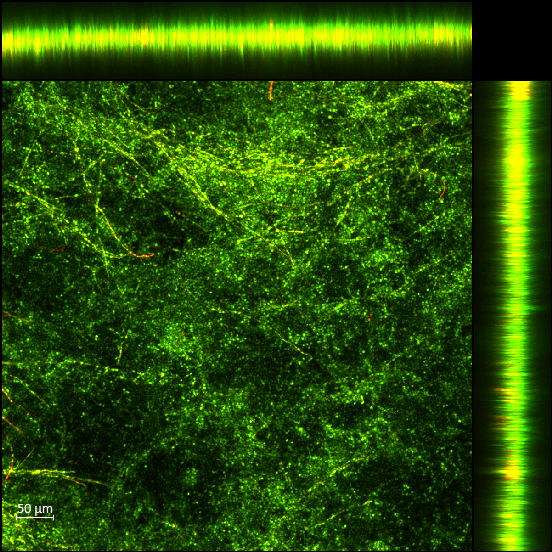

Supplement: S61 Fig — (TIFF) [file pone.0292430.s061.tiff]

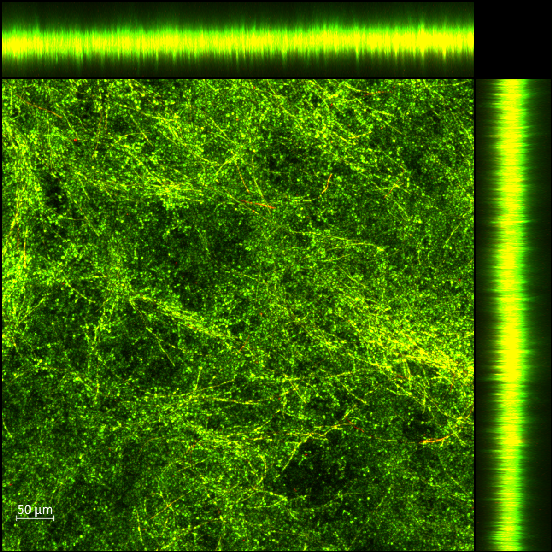

Supplement: S62 Fig — (TIFF) [file pone.0292430.s062.tiff]

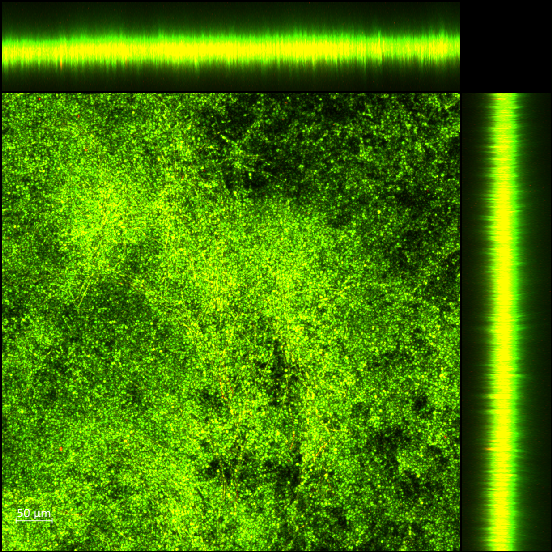

Supplement: S63 Fig — (TIFF) [file pone.0292430.s063.tiff]

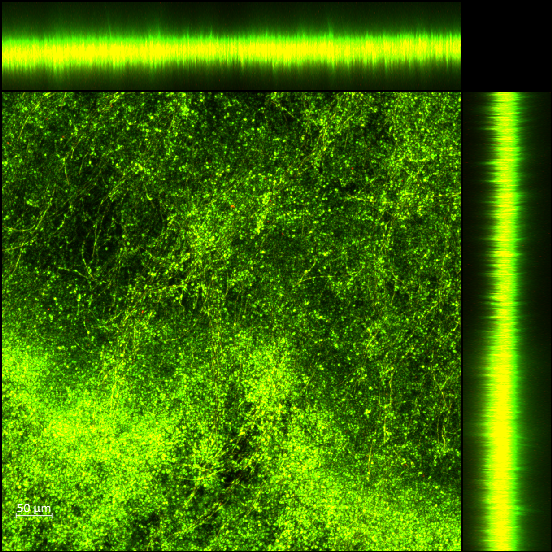

Supplement: S64 Fig — (TIFF) [file pone.0292430.s064.tiff]

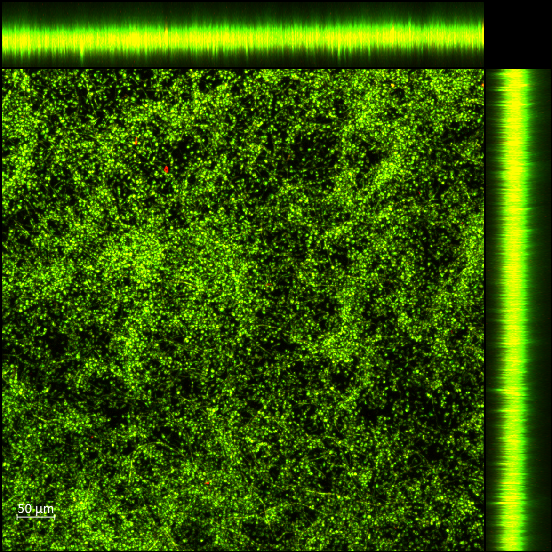

Supplement: S65 Fig — (TIFF) [file pone.0292430.s065.tiff]

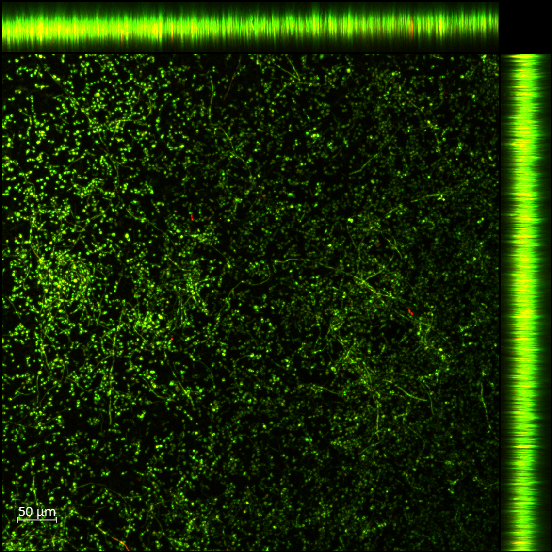

Supplement: S66 Fig — (TIFF) [file pone.0292430.s066.tiff]

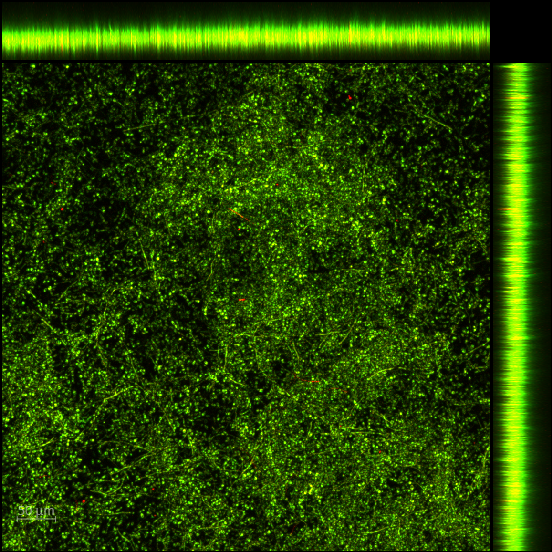

Supplement: S67 Fig — (TIFF) [file pone.0292430.s067.tiff]

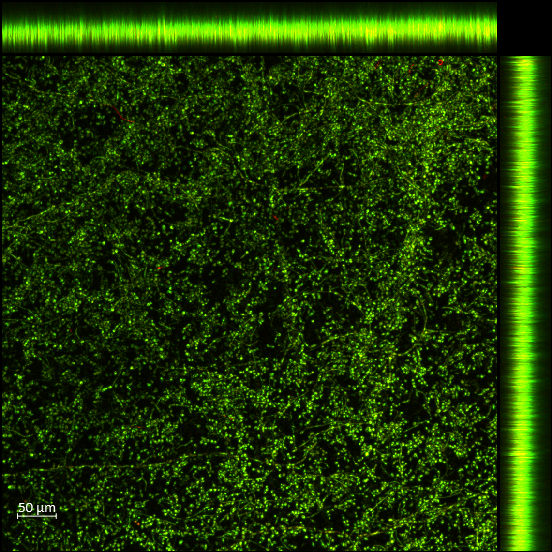

Supplement: S68 Fig — (TIFF) [file pone.0292430.s068.tiff]

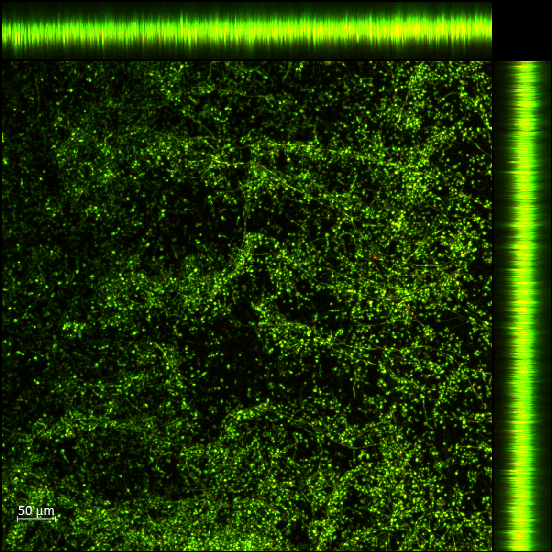

Supplement: S69 Fig — (TIFF) [file pone.0292430.s069.tiff]

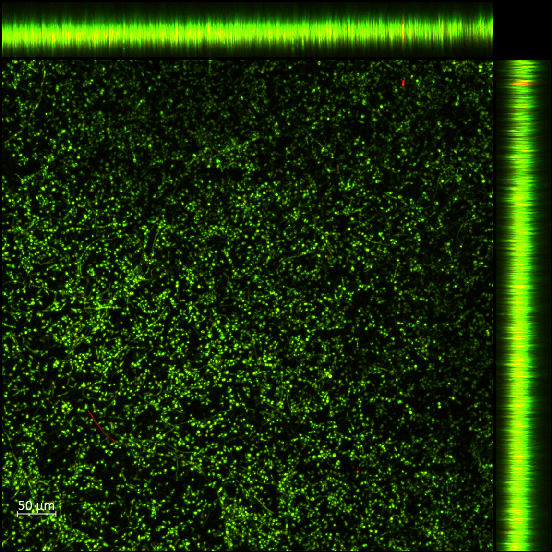

Supplement: S70 Fig — (TIFF) [file pone.0292430.s070.tiff]
