# Supplementary material for: Microbial adhesion and biofilm formation by Candida albicans on 3D-printed denture base resins
Source: PLoS One. 2023 Oct 4;18(10):e0292430. doi: 10.1371/journal.pone.0292430 (PMC10550158; doi:10.1371/journal.pone.0292430)
Supplement: S1 Table — * Image chosen to represent the group in Fig 6. (DOCX) [file pone.0292430.s071.docx]

| Resin | Period | Image | Live | Dead | Thickness |
| --- | --- | --- | --- | --- | --- |
| Lucitone 550 | Adhesion 90min | Fig S1* | 65.272 | 21.038 | 20 |
|  |  | Fig S2 | 38.106 | 12.759 | 20 |
|  |  | Fig S3 | 17.532 | 6.324 | 16 |
|  |  | Fig S4 | 37.033 | 12.208 | 22 |
|  |  | Fig S5 | 63.599 | 21.587 | 22 |
|  |  | Fig S6 | 70.895 | 25.539 | 20 |
|  |  | Fig S7 | 90.320 | 43.830 | 24 |
|  |  | Fig S8 | 87.405 | 42.411 | 24 |
|  |  | Fig S9 | 70.636 | 34.191 | 18 |
|  |  | Fig S10 | 71.844 | 34.901 | 22 |
|  |  | Fig S11 | 51.715 | 23.610 | 18 |
|  |  | Fig S12 | 33.098 | 14.434 | 18 |
|  | Mean |  | 58.12125 | 24.40267 | 20.33333 |
|  | SD |  | 22.65061 | 12.2081 | 2.534609 |
|  | Biofilm 48h | Fig S13 | 75.575 | 38.650 | 52 |
|  |  | Fig S14* | 52.450 | 31.673 | 50 |
|  |  | Fig S15 | 29.961 | 27.835 | 46 |
|  |  | Fig S16 | 37.419 | 20.525 | 44 |
|  |  | Fig S17 | 97.514 | 52.159 | 52 |
|  |  | Fig S18 | 30.906 | 19.154 | 48 |
|  |  | Fig S19 | 51.220 | 23.110 | 48 |
|  |  | Fig S20 | 42.131 | 20.230 | 56 |
|  |  | Fig S21 | 42.202 | 21.023 | 50 |
|  |  | Fig S22 | 39.531 | 18.447 | 42 |
|  |  | Fig S23 | 57.904 | 27.757 | 48 |
|  |  | Fig S24 | 34.742 | 16.710 | 44 |
|  | Mean |  | 49.29625 | 26.43942 | 48.33333 |
|  | SD |  | 19.92085 | 10.30113 | 3.98482 |

Table S1. Descriptive values of the supplementary figures corresponding to the

adhesion and biofilm periods of Lucitone 550 resin.

* Images chosen to represent the groups in Fig. 6.
