# Supplementary material for: Microbial adhesion and biofilm formation by Candida albicans on 3D-printed denture base resins
Source: PLoS One. 2023 Oct 4;18(10):e0292430. doi: 10.1371/journal.pone.0292430 (PMC10550158; doi:10.1371/journal.pone.0292430)
Supplement: S2 Table — * Image chosen to represent the group in Fig 6. (DOCX) [file pone.0292430.s072.docx]

Table S2. Descriptive values of the supplementary figures corresponding to the

adhesion and biofilm periods of Cosmos Denture resin.

| Resin | Period | Image | Live | Dead | Thickness |
| --- | --- | --- | --- | --- | --- |
| Cosmos Denture | Adhesion 90min | Fig S25 | 94.291 | 44.298 | 26 |
|  |  | Fig S26* | 96.147 | 44.789 | 26 |
|  |  | Fig S27 | 84.078 | 38.107 | 26 |
|  |  | Fig S28 | 82.162 | 36.505 | 28 |
|  |  | Fig S29 | 77.996 | 33.785 | 28 |
|  |  | Fig S30 | 80.275 | 35.432 | 30 |
|  |  | Fig S31 | 153.967 | 66.974 | 39 |
|  |  | Fig S32 | 145.008 | 60.788 | 36 |
|  |  | Fig S33 | 162.469 | 67.717 | 33 |
|  |  | Fig S34 | 150.136 | 60.880 | 30 |
|  |  | Fig S35 | 79.552 | 31.893 | 27 |
|  |  | Fig S36 | 112.631 | 40.793 | 28 |
|  | Mean |  | 109.8927 | 46.83008 | 29.75 |
|  | SD |  | 33.37096 | 13.44076 | 4.202272 |
|  | Biofilm 48h | Fig S37 | 50.082 | 21.363 | 56 |
|  |  | Fig S38 | 60.391 | 25.743 | 52 |
|  |  | Fig S39* | 80.645 | 39.526 | 52 |
|  |  | Fig S40 | 76.838 | 35.889 | 52 |
|  |  | Fig S41 | 50.362 | 23.292 | 54 |
|  |  | Fig S42 | 60.017 | 32.043 | 67 |
|  |  | Fig S43 | 106.354 | 55.153 | 56 |
|  |  | Fig S44 | 140.029 | 80.739 | 62 |
|  |  | Fig S45 | 119.233 | 63.152 | 58 |
|  |  | Fig S46 | 50.355 | 22.517 | 52 |
|  | Mean |  | 79.4306 | 39.9417 | 56.1 |
|  | SD |  | 32.11191 | 20.11388 | 5.043147 |

* Image chosen to represent the group
