# Supplementary material for: Microbial adhesion and biofilm formation by Candida albicans on 3D-printed denture base resins
Source: PLoS One. 2023 Oct 4;18(10):e0292430. doi: 10.1371/journal.pone.0292430 (PMC10550158; doi:10.1371/journal.pone.0292430)
Supplement: S3 Table — * Image chosen to represent the group in Fig 6. (DOCX) [file pone.0292430.s073.docx]

Table S3. Descriptive values of the supplementary figures corresponding to the

adhesion and biofilm periods of NextDent Denture 3D+ resin.

| Resin | Period | Image | Live | Dead | Thickness |
| --- | --- | --- | --- | --- | --- |
| NextDent Denture 3D+ | Adhesion 90min | Fig S47 | 89.878 | 34.286 | 24 |
|  |  | Fig S48 | 97.485 | 39.312 | 24 |
|  |  | Fig S49 | 98.902 | 41.202 | 24 |
|  |  | Fig S50 | 103.957 | 43.397 | 22 |
|  |  | Fig S51* | 111.052 | 46.330 | 26 |
|  |  | Fig S52 | 102.783 | 43.259 | 26 |
|  |  | Fig S53 | 143.709 | 60.875 | 28 |
|  |  | Fig S54 | 109.080 | 52.505 | 28 |
|  |  | Fig S55 | 111.035 | 52.247 | 30 |
|  |  | Fig S56 | 113.328 | 54.502 | 30 |
|  |  | Fig S57 | 93.703 | 43.257 | 24 |
|  |  | Fig S58 | 93.273 | 43.197 | 30 |
|  | Mean |  | 105.6821 | 46.19742 | 26.33333 |
|  | SD |  | 14.28711 | 7.441703 | 2.806918 |
|  | Biofilm 48h | Fig S59 | 56.048 | 23.498 | 56 |
|  |  | Fig S60 | 56.248 | 22.457 | 48 |
|  |  | Fig S61 | 74.678 | 33.823 | 54 |
|  |  | Fig S62 | 106.342 | 53.535 | 56 |
|  |  | Fig S63 | 138.920 | 72.158 | 54 |
|  |  | Fig S64 | 119.741 | 61.684 | 58 |
|  |  | Fig S65* | 83.687 | 45.547 | 52 |
|  |  | Fig S66 | 33.532 | 16.729 | 46 |
|  |  | Fig S67 | 59.563 | 30.694 | 48 |
|  |  | Fig S68 | 46.363 | 23.792 | 42 |
|  |  | Fig S69 | 46.603 | 24.313 | 46 |
|  |  | Fig S70 | 38.264 | 18.897 | 44 |
|  | Mean |  | 71.66575 | 35.59392 | 50.33333 |
|  | SD |  | 33.92065 | 18.30505 | 5.31436 |

* Image chosen to represent the group
